# AI-driven 3D bioprinting for regenerative medicine: From bench to bedside

**DOI:** 10.1016/j.bioactmat.2024.11.021

**Published:** 2024-11-23

**Authors:** Zhenrui Zhang, Xianhao Zhou, Yongcong Fang, Zhuo Xiong, Ting Zhang

**Affiliations:** a Biomanufacturing Center, Department of Mechanical Engineering, Tsinghua University, Beijing, 100084, PR China; b Biomanufacturing and Rapid Forming Technology Key Laboratory of Beijing, Beijing, 100084, PR China; c “Biomanufacturing and Engineering Living Systems” Innovation International Talents Base (111 Base), Beijing, 100084, PR China; d State Key Laboratory of Tribology in Advanced Equipment, Tsinghua University, Beijing, 100084, PR China

**Keywords:** 3D bioprinting, Artificial intelligence, Machine learning, Quality by design, Regenerative medicine, Clinical translation

## Abstract

In recent decades, 3D bioprinting has garnered significant research attention due to its ability to manipulate biomaterials and cells to create complex structures precisely. However, due to technological and cost constraints, the clinical translation of 3D bioprinted products (BPPs) from bench to bedside has been hindered by challenges in terms of personalization of design and scaling up of production. Recently, the emerging applications of artificial intelligence (AI) technologies have significantly improved the performance of 3D bioprinting. However, the existing literature remains deficient in a methodological exploration of AI technologies' potential to overcome these challenges in advancing 3D bioprinting toward clinical application. This paper aims to present a systematic methodology for AI-driven 3D bioprinting, structured within the theoretical framework of Quality by Design (QbD). This paper commences by introducing the QbD theory into 3D bioprinting, followed by summarizing the technology roadmap of AI integration in 3D bioprinting, including multi-scale and multi-modal sensing, data-driven design, and in-line process control. This paper further describes specific AI applications in 3D bioprinting's key elements, including bioink formulation, model structure, printing process, and function regulation. Finally, the paper discusses current prospects and challenges associated with AI technologies to further advance the clinical translation of 3D bioprinting.

## Highlights

• AI-driven QbD can enhance 3D bioprinting's quality, rapidity, economy and scalability.
• AI integrated in 3D bioprinting enables multi-scale and multi-modal sensing, data-driven design, and in-line process control.
• Promising development directions of AI technology are proposed to further advance clinical translation of 3D bioprinting.

## Graphical abstract

**Figure:**
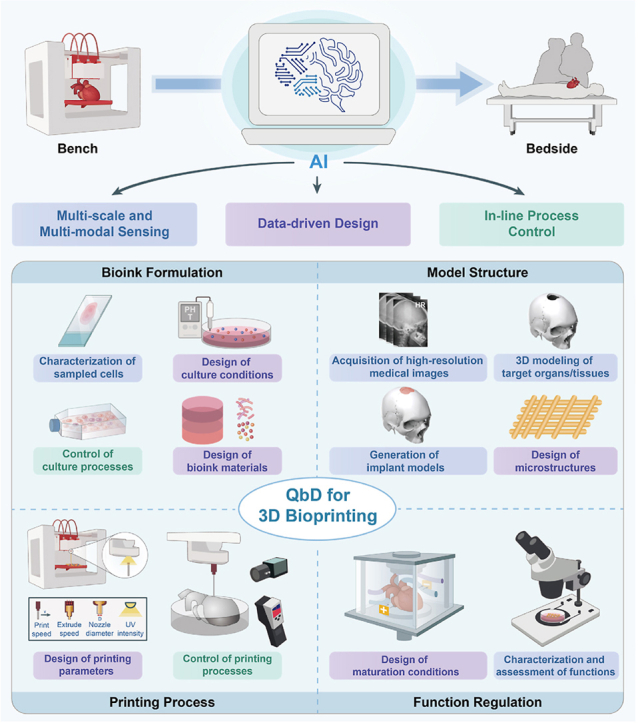

## 1 Introduction

3D bioprinting technology can be explored to fabricate well-defined multi-scale structures by precisely manipulating biomaterials and cells within three-dimensional space [[1], [2], [3]]. In the field of regenerative medicine, 3D bioprinted products (BPPs) can be used as patient-specific implants for regenerative repair of damaged organs/tissues or as patient-specific in vitro models for disease modeling and drug screening [4,5]. Despite recent progress in 3D bioprinting technology, clinical cases of BPPs applied in humans remain scarce. We identify several challenges at the R&D and production stages that hinder 3D bioprinting's clinical translation:

(ⅰ) **Personalization of design:** The BPPs for clinical practice should be patient-specific [6], due to the immunity-, tissue-, structure-, and function-specific nature of repaired parts [[7], [8], [9]]. This necessitates that the design of BPPs replicates the complexity and specificity of natural tissues across multi-materials and multi-scale structures. In this regard, optimized design ensuring effectiveness introduces an extensive range of design parameters, requiring significant trial and error. However, because of the large differences and small batches of BPPs, it is difficult to amortize the R&D costs, resulting in the contradiction of “effectiveness-economy” [9].
(ⅱ) **Scaling up of production:** Considering regulation, international regulatory frameworks for the commercialization of a medical device or an Advanced Therapeutic Medicinal Product (ATMP) require strict quality control to ensure that BPPs are manufactured in a reproducible and contamination-free manner [10]. However, current BPPs are typically designed and produced by skilled researchers in academic laboratories, which involves a number of complex manual operations. As a result, BPPs are small-scale, poorly repeatable, expensive, and difficult to regulate [6,9].

Therefore, to facilitate the clinical translation of BPPs, it is essential to enhance quality in both the R&D and production stages. The traditional Quality by Testing approach emphasizes post-production testing, which is impractical for the clinical application of BPPs, as changes in the clinical stage are costly and difficult [6]. Furthermore, Quality by Testing typically focuses on optimizing individual variables, making it inadequate for addressing the multi-material and multi-scale design requirements of BPPs. To address the above deficiencies, Quality by Design (QbD) is a promising solution for BPPs requiring effectiveness, economy, and regulatory compliance. This approach has been widely adopted by the U.S. FDA to enhance quality and efficiency, as well as to reduce costs and regulatory burdens, in fields related to 3D bioprinting such as biopharmaceuticals [11]. Compared to Quality by Testing, QbD posits that all problems affecting the quality of the final product are related to its design. Accordingly, in the beginning R&D stage, products should be designed correctly considering quality optimization. In the production stage, QbD uses process control to develop robust and reliable production procedures based on an in-depth understanding of products and processes. Currently, the introduction of QbD into 3D bioprinting is currently being discussed by the academic community, industry, and government [[12], [13], [14]].

In the field of 3D bioprinting, Artificial intelligence (AI), represented by machine learning (ML), has seen widespread application [15]. This revolutionary technology holds great potential in accelerating the deployment of QbD in 3D bioprinting. For example, deep learning can be used to automatically acquire critical quality attributes of BPPs from various sensor data, eliminating the need for extensive manual characterizations and thereby reducing costs. Supervised learning can be used to model the complex mapping relationship between critical material attributes/process parameters and critical quality attributes of BPPs. Given the vast number of design parameters, this approach significantly reduces the need for trial-and-error experiments. Reinforcement learning can be used to construct control strategies of 3D bioprinting, adapting to dynamic working scenarios based on interactions with the environment to meet the needs of scaling-up production. To summarize, AI-driven QbD will accelerate the translation of 3D bioprinting from bench to bedside [4,[16], [17], [18]].

Although recent review papers have elaborated on the utilization of AI in 3D bioprinting [10,[19], [20], [21], [22]], most take a workflow-centric approach, primarily summarizing the specific applications of AI in various steps of 3D bioprinting. In contrast, this paper adopts a clinical product perspective, leveraging the QbD theory from industrial production to propose a systematic framework for applying AI to 3D bioprinting. We begin by analyzing the fundamental methodologies and the technology roadmap of integrating AI with 3D bioprinting within the QbD framework, focusing on multi-scale and multi-modal sensing, data-driven design, and in-line process control. Next, we explore the current research status and application potential of AI across key elements of 3D bioprinting, including bioink formulation, model structure, printing process, and function regulation. Lastly, we propose future directions and challenges for AI in 3D bioprinting. We believe our theoretical framework will further guide the application of AI in broader fields such as tissue engineering, biofabrication, and related domains. We hope this review enables AI scientists to more effectively engage with the 3D bioprinting field, while helping 3D bioprinting researchers deepen their understanding of AI technologies and adopt the latest advancements.

## 2 AI-driven QbD framework and roadmap for 3D bioprinting

**Fig. 1:**
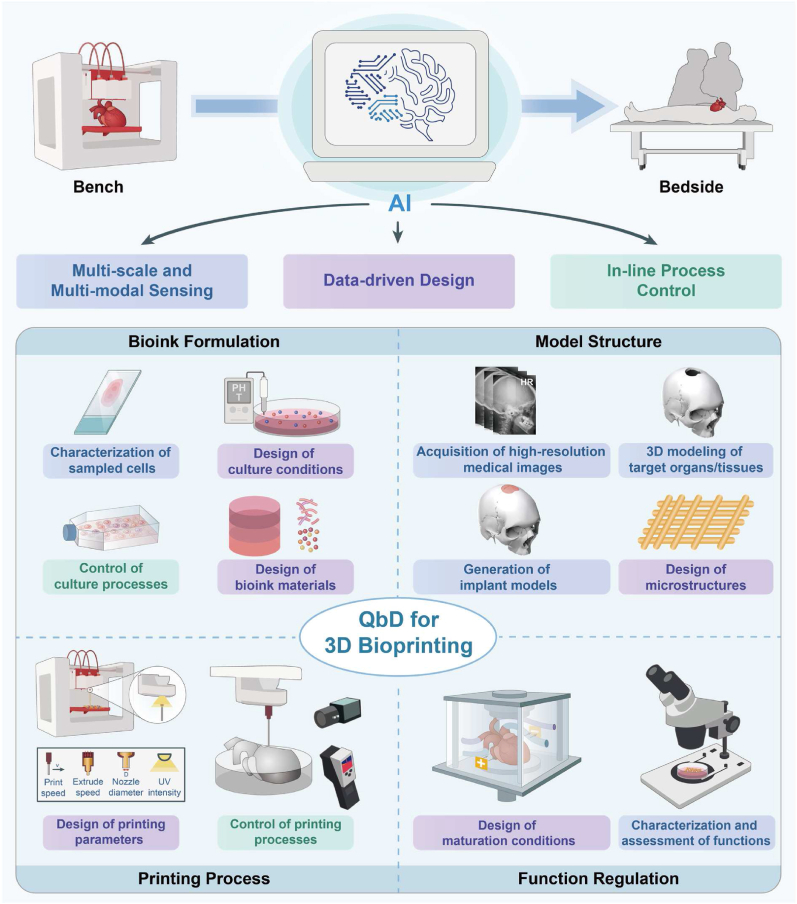
Roadmap of AI-driven QbD for 3D bioprinting, containing multi-scale and multi-modal sensing, data-driven design, and in-line process control, which can be used in four key elements, including bioink formation, model structure, printing process and function regulation.

3D bioprinting comprises four key elements: bioink formulation, model structure, printing process, and function regulation. Each element consists of multiple unit operations (UOs) [[23], [24], [25]] within which AI-driven QdD can be integrated, such as design of bioink materials, design of microstructures, control of printing processes, and characterization and assessment of functions (Fig. 1). This chapter aims to offer a comprehensive analysis framework outlining the primary application scenarios of AI technology in 3D bioprinting. Specific applications of AI technology within separate UOs will be detailed in Chapters 3, 4, 5, and 6. Additionally, focusing on the application of AI technologies in 3D bioprinting, this paper has not included a detailed introduction to basic methods and concepts of AI technologies, which can be found in other references [26].

### 2.1 QbD theory for 3D bioprinting

Given the inherent complexity of original QbD theories, this section simplifies the relevant terms and concepts, with further details of QbD available in other specialized references [[27], [28], [29], [30], [31], [32], [33]]. Following this, integrating 3D bioprinting, we provide a detailed explanation of the simplified QbD terminology:

(ⅰ) **Critical quality attributes (CQA):** CQA refer to a physical, chemical, or biological property that reflects product quality [33]. In this regard, we propose two approaches: the forming-based and the function-based (Table 1). The forming-based CQA of BPPs are directly evaluated by their printability, which can be predicted by the rheological and gelation properties of bioinks. Printability is a critical category of CQA for mimicking functions of natural organs/tissues, as geometry profoundly determines mechanical and biological properties [[34], [35], [36]]. Further details on printability can be found in other comprehensive reviews [37]. The function-based CQA mainly include transport, mechanical, and biological properties. The transport properties are crucial for the survival and functionalization of BPPs, which ensure the delivery of oxygen, nutrients, biological factors, and drugs, as well as the removal of metabolites [38]. Moreover, it is imperative to form effective vascular networks in large-scale thick tissues [39]. BPPs should possess mechanical responses matching those of natural tissues as well as suitable degradation and swelling properties for in vitro culture and in vivo implantation [40]. The biological properties of BPPs can be assessed at various levels, including tissue, cell, and gene expression. Further details on biological properties can be found in other comprehensive reviews [41].

**Table 1:** Examples of CQA in 3D bioprinting.

Approach	Examples of CQA	
Forming-based	Printability	Extrudability, filament formation, shape fidelity
	Rheological properties	Shear-thinning, viscoelasticity, yield stress, constitutive model
	Gelation properties	Gelation time, gel fraction
Function-based	Transport properties	Effective mass diffusion rate, vascularization
	Mechanical properties	Mechanical response: Young's modulus, response curve, constitutive model, strength
		Degradation properties: degradation rate
		Swelling properties: swelling rate
Function-based	Biological properties [41]	Gene expression: cell differentiation and phenotype, cell health, genomic stability
		Protein expression: matrix production, cell differentiation and phenotype, cell health
		Cell metabolism: nutrient and waste analysis, cell signaling, cellular products, cell health
		Cell properties: viability, morphology, motility, confluence, cell number, cell health
		Tissue properties: morphology, function
(ⅱ) **Critical material attributes (CMA)/Critical process parameters (CPP):** CMA and CPP respectively refer to material attributes and process attributes that have a significant impact on product CQA [33]. For example, in the design of culture conditions, the compositions of the culture medium serve as CMA, and the culture process parameters serve as CPP. In the design of printing parameters, the bioink formulations serve as CMA, and the printing process parameters serve as CPP.
(ⅲ) **Design space:** In QbD, CQA are determined by CMA and CPP. In this context, the design space describes the distribution of CQA under combinations of CMA/CPP within a certain range. In low-dimensional cases, the design space can be visualized in the form of phase diagrams or process windows, serving as a guide for designing CMA/CPP.
(ⅳ) **Control strategy:** Control strategy refers to a planned set of controls over CMA/CPP, derived from product and process understanding, ensuring CQA of the production process [33].
(ⅴ) **Risk Assessment:** Risk assessment refers to a process of quality risk management that can identify the impact of individual CMA/CPP on product CQA and the interactions among CMA/CPP [33]. This process enables a deeper understanding of the underlying process mechanisms.

### 2.2 Roadmap of AI-driven QbD for 3D bioprinting

Within the QbD framework, AI technologies enable the faster, more economical, and more scalable design and production of BPPs with higher CQA. This helps address the challenges of personalized design and scaling-up production in 3D bioprinting, accelerating the translation from bench to bedside. Here, we discuss the roadmap of AI technology for 3D bioprinting from three dimensions:

(ⅰ) **Multi-scale and multi-modal sensing:** The structural and functional features across various scales are extracted by diverse sensors to rapidly and economically acquire CQA, CMA, and CPP.
(ⅱ) **Data-driven design:** The intricate relationship between CMA/CPP and CQA is modeled through data to precisely determine the optimal design space.
(ⅲ) **In-line process control:** The control procedures of process quality are then implemented through the established control strategy, which integrates AI technology in the former two dimensions.

In 3D bioprinting, each UO corresponds to the application of AI technology in one of the aforementioned three dimensions (Fig. 1). Thus, it is crucial to clarify the application scope of QbD-related terms. For the latter two dimensions, the corresponding UOs create products (such as cells, printed models, and BPPs), thus defining the application scope of QbD-related terms as the UOs themselves. For the first dimension, the corresponding UOs aim to determine the CQA, CMA, and CPP of products from other UOs, thus defining the application scope of QbD-related terms as their associated products.

#### 2.2.1 Multi-scale and multi-modal sensing

**Fig. 2:**
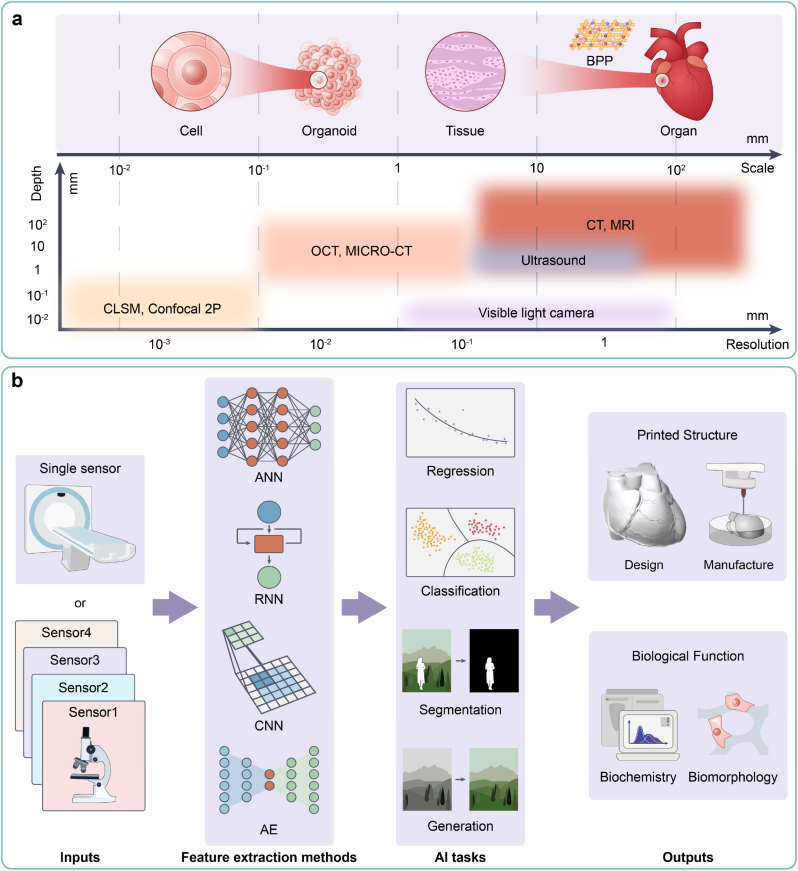
Multi-scale and multi-modal sensing. (a) A “scale-depth-precision” contradiction of imaging technologies. (b) A pipeline of AI-driven multi-modal sensing methodologies to obtain comprehensive results.
(ⅱ) **Insufficient information abundance:** Complex objects encompass multiple attributes, yet single sensing modalities excel in detecting specific attributes only, resulting in difficulties in precisely sensing multiple attributes simultaneously and biased sensing results.
(ⅲ) **Low automation:** Traditional sensing methodologies rely on skilled operators to perform tedious manual operations with professional equipment and reagents in the pre-sensing and post-sensing stages. This reliance results in poor economy and rapidity. Moreover, manual operations entail subjective errors, contamination risks, and scaling-up challenges, further impeding repeatability, safety, and scalability.

In 3D bioprinting, each UO integrates various sensors to capture multi-modal data, facilitating the acquisition of multi-scale information crucial for personalized design and scaling-up production. The sensing process typically encompasses three sequential stages: (ⅰ) pre-sensing, involving pre-processing of the sensed object, such as tissue section preparation and staining; (ⅱ) sensing, entailing the utilization of various sensors to measure specific attributes of the sensed object and generating corresponding sensor data, and (ⅲ) post-sensing, involving processing and analyzing the collected sensor data to derive quantitative sensing results, including CQA, CMA, and CPP. Traditional sensing methodologies exhibit deficiencies in precision, rapidity, economy, repeatability, safety, and scalability, thereby impeding the clinical translation of BPPs. We attribute these deficiencies primarily to the following three key factors:

(ⅰ) **“Scale-depth-precision” contradiction:** Imaging, as the primary sensing methodology [42]*,* can sense objects in 3D bioprinting spanning scales from micrometers to centimeters [43]. Typically, larger objects necessitate greater imaging depth but exhibit lower resolution, and vice versa, hindering the extraction of detailed information of large-scale objects and 3D spatial information of small-scale objects (Fig. 2a).

AI technology, particularly deep learning methodologies, presents viable solutions to the above challenges [41,44]. Based on input data from diverse sensors, specific feature extraction methods such as artificial neural networks (ANNs), convolutional neural networks (CNNs), recurrent neural networks (RNNs), and auto-encoders (AEs) are deployed to execute distinct AI tasks, including regression, classification, segmentation, and generation. Consequently, during the 3D bioprinting process, AI-driven sensing yields comprehensive results encompassing aspects such as the design and manufacturing of printed structures, as well as biochemical and morphological functions (Fig. 2b).

AI technology serves as a remedy for the contradiction of “scale-depth-precision,” enabling precise imaging of objects across various scales. In the realm of large-scale objects, as encountered in medical imaging modalities such as computed tomography (CT) and magnetic resonance imaging (MRI), AI-based super-resolution and denoising can enhance image resolution [45]. Super-resolution enhances overall image clarity, whereas denoising mitigates artifacts induced by patients' motion. Conversely, in the realm of small-scale objects, AI technology facilitates the extraction of 3D spatial information. Notably, studies have demonstrated the efficacy of AI-based automatic segmentation and 3D reconstruction in elucidating the spatial structure of minute tissues from serial sections [46], as well as the spatial distribution of the nucleus from confocal laser scanning microscopy (CLSM) images [47].

AI technology has the capacity to significantly augment information abundance and achieve precise and robust sensing of complex objects. Multi-modal machine learning (MML) stands as a prime example, integrating attribute information gathered from diverse sensors to markedly enhance the sensing precision of complex objects. Numerous studies have explored the application of MML across fields pertinent to 3D bioprinting. Examples include the segmentation of soft tissue sarcomas utilizing four types of medical images including CT, T1 MRI, T2 MRI, and Positron Emission Tomography (PET) [48], virtual staining of tissue sections through non-linear multi-modal imaging (NLM) [49], and monitoring of the printing process leveraging three types of sensor data (layerwise electro-optical, acoustic, and multispectral), alongside off-line process parameters [50].

AI technology can extract complex features and recognize complex patterns to reduce and replace manual operations. On the one hand, AI technology can emulate humans' sensing patterns, replacing humans in repetitive and labor-intensive tasks automatically, such as segmenting medical images [44] and assessing the matching degree of CMA/CPP during the printing process. On the other hand, AI technology can recognize patterns that are challenging for humans to learn from physical phenomena, thereby simplifying the sensing process and manual operations and minimizing reliance on expensive equipment and reagents. For instance, virtual staining technology [[51], [52], [53]] can comprehend the transformation patterns between different stained images, thus eliminating the need for multiple dyeing operations. The “digital rheometer twins” can derive rheological constitutive models from rheological data, reducing the dependence on rheometers [54]. In essence, the replacement and reduction of manual operations by AI technology serve to mitigate subjective errors, contamination risks, and costs while simultaneously enhancing repeatability, safety, and economy. Moreover, automatic sensing processes contribute to enhanced rapidity and scalability.

#### 2.2.2 Data-driven design

**Fig. 3:**
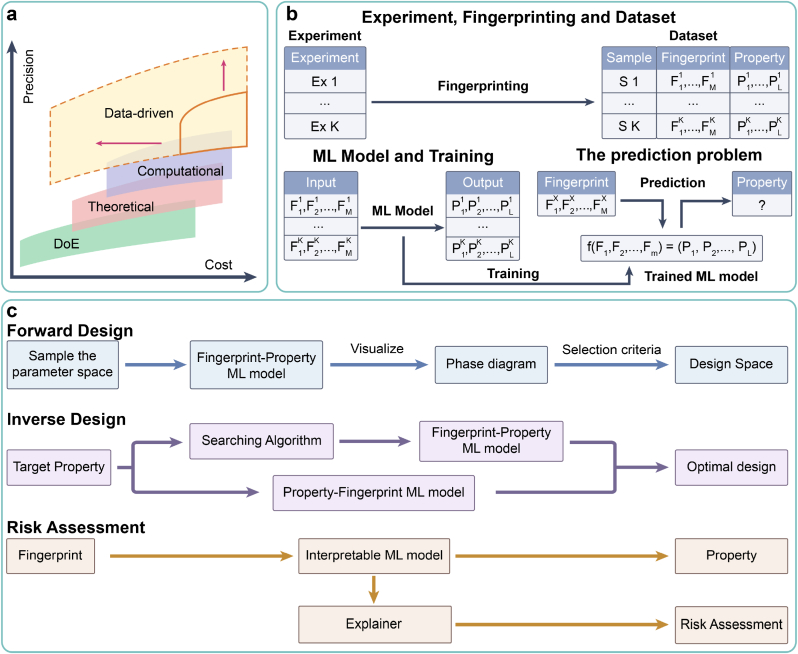
Data-driven design. (a) A “precision-cost” landscape of four modeling paradigms. (b) A typical workflow of ML-based data-driven paradigm. (c) Three main ML tasks within data-driven design.

The core principle of QbD asserts that CQA is contingent upon CMA/CPP. Consequently, the procedural framework for personalized design within QbD can be succinctly outlined as follows: (ⅰ) modeling of the potential mapping relationship between CMA/CPP and CQA, (ⅱ) determination of the optimal design space of CMA/CPP, taking enhancement of CQA as the primary objective, and (ⅲ) risk assessment to scrutinize the effect of each CMA/CPP on CQA. In the field of 3D bioprinting, personalized design involves various objects, including culture conditions for sampled cells, bioink materials, microstructures of printed models, printing parameters, and maturation conditions of BPPs (Fig. 1). Notably, in tackling modeling problems, the dilemma of “precision-cost” frequently arises [55]. As model precision (or problem complexity) escalates, a corresponding increase in associated costs (such as financial investment, time, and human resources) is observed, while the marginal precision incrementally diminishes (Fig. 3a).

Presently, four modeling paradigms have emerged, including the design-of-experiment (DoE), theoretical, computational, and data-driven paradigms (Fig. 3a) [26,56]. Although widely applicable, the DoE paradigm requires a substantial number of manual experiments to traverse the parameter space [57], resulting in labor-intensive processes. Additionally, the conventional response surface methodology for DoE has a limited ability to model complex relationships. To augment precision and mitigate the necessity for manual experiments, theoretical and computational paradigms have been developed. Both paradigms construct mathematical models based on domain knowledge (such as physics and biology) to expound process mechanisms, offering a “white box” effect [56]. The disparity lies in the approach. The theoretical paradigm entails the manual construction of theoretical formulas, providing prediction with faster speed but lower precision. In contrast, the computational paradigm relies on numerical simulations, such as finite element analysis (FEA) and computational fluid dynamics (CFD). This paradigm necessitates substantial computational resources, providing prediction with higher precision but slower speed. With advancements in computational precision, numerical simulations are progressively superseding manual experiments.

The aforementioned three paradigms have achieved certain advancements in the field of 3D bioprinting [[58], [59], [60], [61], [62]]. However, as 3D bioprinting advances towards clinical translation, especially in constructing substitutes of natural tissues/organs, the demand for higher modeling precision continues to increase. We identify three key dimensions that increasingly highlight the inherent complexity of 3D bioprinting:

(ⅰ) **Multi-domain fusion:** 3D bioprinting necessitates the integration of knowledge spanning diverse domains, including biology, machinery, materials, and medicine. Constructing mathematical models in the theoretical and computational paradigms has proven challenging due to this multi-disciplinary nature.
(ⅱ) **Multi-scale coexistence:** Within the realm of 3D bioprinting, factors such as CQA, CMA, and CPP operate across multiple scales. These scales encompass nano scale (such as molecular fragments of bioink materials), micro scale (such as microstructures of printed models), and macro scale (such as mechanical properties of BPPs). The theoretical and computational paradigms encounter difficulties in addressing these multi-scale modeling problems due to a dearth of constitutive models and the burden of excessive computational loads [63].
(ⅲ) **Multi-property coupling:** For certain design objects within 3D bioprinting, such as bioink materials and microstructures of printed models, conflicting property requirements arise for design parameters. Examples include printability versus biocompatibility necessitating considerations of viscosity [40] and stiffness versus transport properties necessitating considerations of porosity [64]. Furthermore, certain CMA/CPP exhibit coupling, such as extrusion speed versus printing speed. These complexities yield a narrow feasible design space and impose stringent requirements on modeling precision.

Given the complexity of these challenges and the cost constraints, traditional paradigms face bottlenecks and are transitioning toward the data-driven paradigm based on machine learning (ML).

The ML-based data-driven paradigm typically employs supervised learning approaches, which can be succinctly defined as constructing a generalizable mapping model between input fingerprints and output properties (Fig. 3b). It primarily includes three key steps [57]:

(ⅰ) **Fingerprinting:** The fingerprints (serving as CMA/CPP) and properties (serving as CQA) of samples are digitally represented, and a structured dataset is constructed. Fingerprinting typically requires domain knowledge and can be conducted manually or automatically (see Section 2.2.1 for details of automatic fingerprinting). Depending on the research objectives, fingerprints can be defined at various scales. Generally, smaller-scale fingerprints entail higher costs for the construction of datasets and ML models, but provide deeper insights, and vice versa.
(ⅱ) **Training:** The mapping model between input fingerprints and output properties is established, predominantly through supervised learning methods such as support vector machines (SVMs), random forests (RFs), k-nearest neighbors (KNNs), and artificial neural networks (ANNs).
(ⅲ) **Prediction:** Following training, the ML model can output the corresponding predicted property for any input fingerprints.

In the context of personalized design within QbD, we summarize three primary tasks for machine learning (Fig. 3c):

(ⅰ) **Forward design:** In scenarios where the parameter space is low-dimensional, a forward design approach is effective, where the candidate CMA/CPP serve as inputs and the predicted CQA serve as outputs. Following training, forward design (or fingerprint-property) ML models using fingerprints as inputs and properties as outputs, can predict the property distribution of the design parameter space through traversal, generating visual representations such as process phase diagrams (or windows) [65]. Through visualization, suitable design space meeting property requirements can be determined.
(ⅱ) **Inverse design** [57]**:** Conversely, in scenarios where the parameter space is high-dimensional, an inverse design approach is preferable where the expected CQA serve as inputs and the recommended CMA/CPP serve as outputs. To address the challenge of multi-property coupling in 3D bioprinting, Pareto optimal combinations of design parameters can be identified using multi-objective optimization techniques [66]. To this end, two solutions are proposed: ⅰ) forward-design models are initially trained, followed by the utilization of heuristic intelligent algorithms such as genetic algorithms to search for the optimal design parameters; ⅱ) inverse design (or property-fingerprint) ML models using properties as inputs and fingerprints as outputs, are directly designed and trained, such as generative ML models based on AEs [67,68] and generative adversarial networks (GANs) [69].
(ⅲ) **Risk assessment:** Unlike the “black-box” modeling of traditional machine learning approaches, interpretable machine learning approaches offer a “white-box” effect. Specifically, following training, interpretable ML models use explainers to quantify the impact of each fingerprint on the property, as well as the interactions between fingerprints [70,71]. This interpretability facilitates the risk assessment of CMA/CPP on CQA, enabling a deeper analysis of process mechanisms, such as the printing process and function regulation [72].

#### 2.2.3 In-line process control

To ensure the effectiveness and economy of BPPs for clinical application, two primary considerations govern the production process [6]. The first is quality, requiring the production process to consistently meet regulatory requirements, to ensure safety and effectiveness. The second is scalability, requiring an easily scalable production process to enable large-scale production at an affordable cost.

In 3D bioprinting, continuous production is involved in two key UOs: control of culture processes and control of printing processes. Due to interference factors such as the process drift and model error, the CQA may deviate from expectations in the actual production process, if the optimal CMA/CPP derived from off-line design is continuously adopted. This error probability is particularly heightened for organ-scale BPPs, due to the large number of required cells and the extended printing cycle. Additionally, long-term, low-latency, high-precision monitoring and calibration of the production process pose challenges for human intervention. Manual operations relying on experience, struggle to scale rapidly, posing difficulties and expenses in increasing production capacity.

**Fig. 4:**
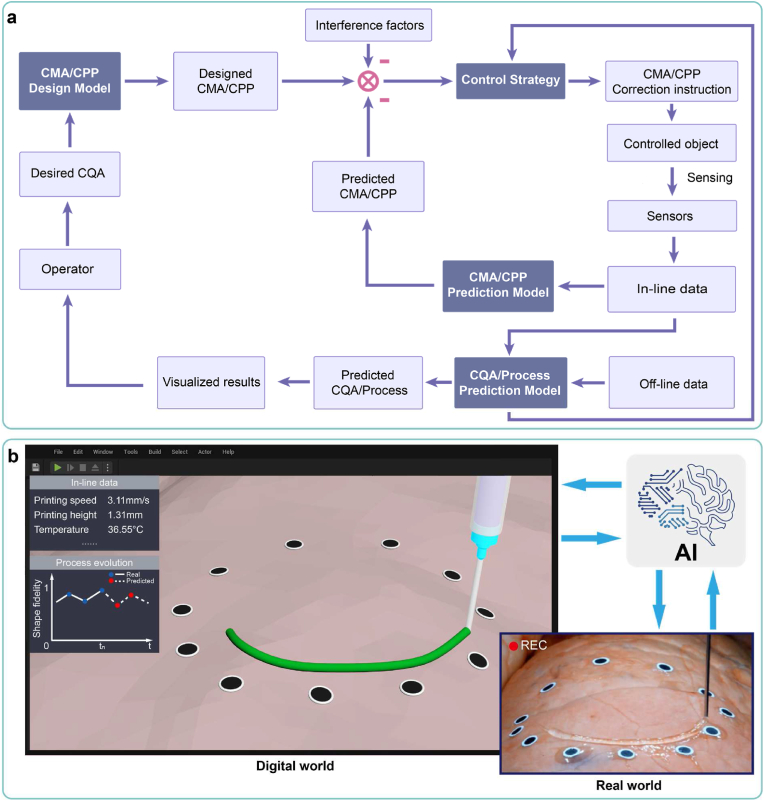
In-line process control. (a) An AI-based in-line process control pipeline, containing four categories of AI models. (b) An illustration of in-line digital twin models for 3D bioprinting, linked to the real production process through monitored data and control commands. Copyright 2020, AAAS.

To address the above problems, based on methodologies in Section 2.2.1, 2.2.2, we propose a general AI-based in-line process control pipeline (Fig. 4a). To maintain the CQA at a high level, the CQA, CMA and CPP are monitored in situ by multiple sensors, and the CMA/CPP is corrected in-line according to the reasonable control strategy. We identify four primary categories of AI models involved in the outlined processes:

(ⅰ) **CMA/CPP design model:** Upon the operator inputting the desired CQA, the AI model outputs the optimal CMA/CPP setting [73].
(ⅱ) **CMA/CPP prediction model:** Various sensors capture in-line sensor data throughout the production process, based on which the AI model assesses the matching degree of current CMA/CPP (such as whether extrusion speed is too fast or slow) that will be transmitted to the control strategy [74].
(ⅲ) **CQA/Process prediction model:** Utilizing the in-line data (such as images and numerical data) and off-line data (or CMA/CPP), the AI model predicts CQA or process evolution, which will be transmitted to the control strategy. Following visualizing, the predicted CQA facilitate defect detection and quality monitoring, supporting for the operator’ decision-making. The predicted process evolution offers the operator early warning of errors and a deeper understanding of process mechanisms.
(ⅳ) **Control Strategy:** The control strategy leverages input information to issue CMA/CPP correction commands, achieving closed-loop correction of the production process. Traditional control strategies rely on rule-based human experience, lacking the ability to learn, which can only address specific and static scenarios [74]. By contrast, the reinforcement learning-based control strategy can learn from interactions with the environment to adapt to complex and dynamic scenarios [75]. The reinforcement learning-based control strategy sets the reward value based on CQA, and takes information such as the matching degree of CMA/CPP and predicted CQA as inputs (or environment state) as well as the correction commands of CMA/CPP as outputs (or action). The reinforcement learning model is trained according to the updated environment state, and will be adopted as the control strategy when the reward value reaches highest. The main algorithms of reinforcement learning include deep q-network (DQN), proximal policy optimization (PPO), and deterministic policy gradient (DPG).

In the field of industrial production, a rapidly emerging research focus is digital twins, which refers to a virtual replica of a physical product or process [6]. This trend has also extended to the field of 3D bioprinting, where digital twin-driven 3D bioprinting is becoming a promising direction [76]. By leveraging the AI models described above, we can establish digital twins for the 3D bioprinting process. Leveraging the aforementioned AI models, the process principles of 3D bioprinting in the digital world of computers can be established, to simulate 3D bioprinting in the real world. Through this approach, we can construct a digital twin of 3D bioprinting, which can operate off-line in the digital world and enable in-line operation through real-time data exchange between the real and digital worlds.

In the design stage, off-line digital twin models enable the rapid execution of numerous virtual experiments in the digital world. Consequently, the design and optimization of CMA/CPP can be accomplished with fewer real experiments, thereby mitigating costs and risks. In the production phase, in-line digital twin models are linked with the real production process through monitored data and control commands, aiming to enhance production efficiency and quality (Fig. 4b). By simulating the process evolution and predicting its outcomes in the digital world, a comprehensive understanding is fostered, facilitating continuous process improvement.

## 3 AI-driven approaches for bioink formulation

**Fig. 5:**
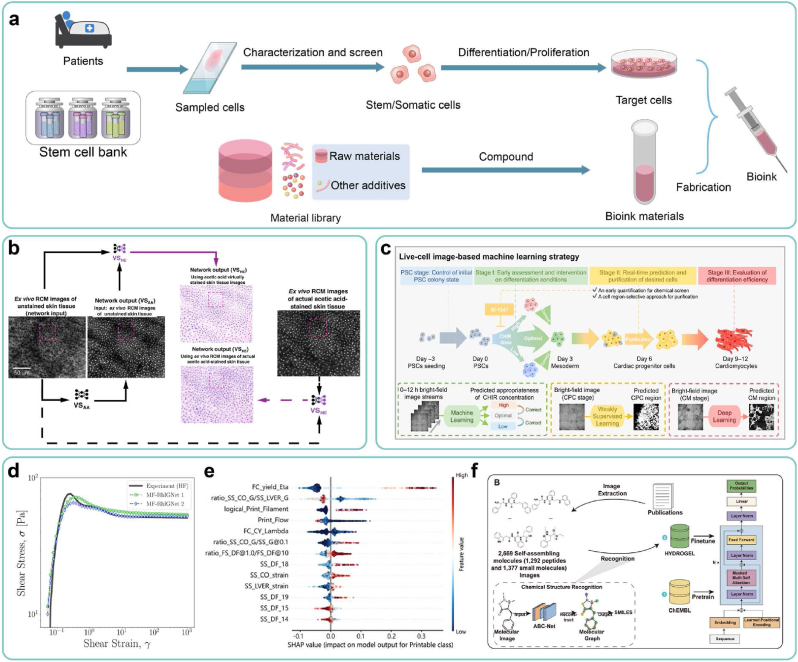
AI-driven approaches for bioink formulation. (a) A pipeline of personalized design of bioinks. (b) Experimental results of virtual staining for salivary gland tissue based on adversarial learning. Copyright 2021, Nature Publishing Group. (c) A workflow of real time monitoring and regulation of PSCs' differentiation process, using multiple AI algorithms. Copyright 2023, Nature Publishing Group. (d) Prediction results of “digital rheometer twins” on rheology of hydrogels. Copyright 2022, PNAS. (e) Risk assessment and mechanism analysis of bioink materials using interpretable ML models. Copyright 2022, Wiley. (f) A general workflow for the design of self-assembling peptide using HydrogelFinder-GPT. Copyright 2024, Wiley.

As the primary element of 3D bioprinting, bioinks serve as a crucial foundation for ensuring the immune, tissue, and function specificity of BPPs. Typically, bioinks contain cells and biomaterials. For cells, the preparation process typically involves: first characterizing cells derived from the patient or shared cell bank and screening suitable ones, as described in Section 3.1; then performing differentiation/proliferation to obtain high-quality cells, as described in Sections 3.2, 3.3. For bioink materials, the formulations are designed aimed at specific properties, as described in Section 3.4. AI technology can be applied to each UO in these processes to accelerate the design and production of personalized bioinks for BPPs (Fig. 5a).

### 3.1 Characterization of sampled cells

Considering the diverse applications of BPPs, selecting appropriate cell sources is a critical factor. Given the inherent immuno-specificity of BPPs, autologous cells derived from the patient are the ideal option. This approach is feasible when fewer cells are required, such as for disease modeling and drug screening. However, for constructing organ-scale in vivo implants, the need for a large number of cells makes this approach less viable. With the continuous advancements in stem cell technology, the issue of immune rejection is being addressed [9]. Several countries and regions have established phenotype-specific stem cell banks [77,78], enabling the rapid provision of large quantities of appropriate cells for patients with varying phenotypes. Allogeneic stem cells obtained through these methods could serve as a new source of cells for constructing organ-scale in vivo implants. Upon selecting the appropriate cell sources, rigorous characterization and screening procedures should be conducted to ensure cell viability and compliance with differentiation and proliferation requirements.

However, conventional destructive characterization methods, such as tissue section preparation and staining, pose numerous challenges in the scaling-up production of cells. Characterized cells often face difficulty undergoing subsequent differentiation, proliferation and other characterizations, which leads to wasting the limited amount of sampled cells and affecting the production efficiency of cells. Furthermore, the extended characterization cycle, spanning from several days to weeks, significantly delays clinical treatment and poses challenges for real-time monitoring of the production process. Additionally, the high cost of specialized equipment and reagents utilized in the characterization process, coupled with labor-intensive and time-consuming manual operations, exacerbates these challenges.

AI-based virtual staining technology offers a solution, enabling non-destructive and rapid characterization of sampled cells [52]. This technology has found application across various organs, such as the liver [[79], [80], [81]], kidney [[82], [83], [84]], stomach [85], and lung [86]. Two types of tasks can be implemented by supervised learning (using paired images for training) or unsupervised learning methods (using unpaired images for training): (ⅰ) generating stained images from the raw images of unstained samples, thus obviating cell-consuming staining procedures [[87], [88], [89]], and (ⅱ) generating diverse and complex staining images from basic staining images, facilitating the characterization of multiple properties through single staining processes [90,91]. For instance, Philip O. Scumpia and Aydogan Ozcan's groups [92] have integrated the aforementioned virtual staining techniques to facilitate the rapid and precise acquisition of multi-modal virtual histology of skin through Al models based on adversarial learning (Fig. 5b). These methods mitigate cell loss, expedite the characterization process, and exhibit significant potential for application in 3D bioprinting.

However, considering the deployment of virtual staining technologies in 3D bioprinting, there are still significant limitations in dataset construction and model evaluation. Regarding datasets, current datasets for virtual staining are primarily focused on pathological/tissue sections, which are insufficient to meet the tailored requirements of 3D bioprinting. For example, the absence of staining data for stem cells and the inability of sectioning methods to provide non-destructive characterization of cell states present significant challenges. Regarding model evaluation, existing evaluation metrics to verify the effectiveness of AI models for virtual staining are mostly based on custom loss functions [92], lacking standardization and generalizability. Considering the safety and regulatory requirements for clinical applications, there is an urgent need to establish a standardized and comprehensive evaluation system to quantitatively assess model performance.

### 3.2 Design of culture conditions

The inter-patient variation of autologous cells significantly surpasses the batch-to-batch variation observed in mature cell lines for laboratory use. Consequently, ensuring cell quality (serving as CQA) necessitates the personalized design of patient-specific media (serving as CMA) and culture process parameters (serving as CPP) [14,93]. Given the intricate composition of the medium, such as carbon sources, amino acids, vitamins and growth factors, which leads to the expansive parameter space, the DoE paradigm encounters challenges [94], whereas the ML-based data-driven paradigm offers substantial advantages [95]. Currently, studies have utilized ML methods to model mapping relationships between media composition [96]/culture process parameters [97] (such as the temperature and duration) and cell quality (such as viability, cell density, and metabolites), accelerating the design of culture conditions. For instance, Dong-Yup Lee's group has utilized the principal component analysis (PCA) algorithm to screen and optimize the culture medium components for Chinese hamster ovary cells, resulting in a 30–40 % improvement in viable cell density during the early growth phase [98].

However, the aforementioned methods necessitate the construction of independent datasets corresponding to specific patients, which proves impractical for the clinical application of 3D bioprinting. The limited quantity of cells sampled from patients hinders high-throughput dataset generation. Meanwhile, since the time-intensive nature of individual culture experiments (usually taking several days), it's also unfeasible to construct datasets in a low-throughput manner, failing to meet clinical urgency. To address the aforementioned issue, a promising solution is to develop patient-universal ML models untied to specific patients, using electronic health records (EHRs) containing characteristics of the patients themselves as inputs. Trained by a large dataset of patients' EHRs, the ML model can quickly output personalized culture conditions when new patients' EHRs are input, eliminating the need for patient-specific cell experiments. This paradigm has already been widely applied in clinical diagnosis and treatment [99].

### 3.3 Control of culture processes

Due to factors such as process drift and model error, deviations in cell quality (serving as CQA) may arise during the actual culture process, if the optimal culture conditions (serving as CMA/CPP) derived from off-line design are continuously employed. Hence, implementing in situ monitoring and in-line correction becomes imperative for maintaining cell quality [14,42]. Deep learning-based label-free detection technology, in the form of CQA prediction models, serves to sense cell quality throughout the culture process, both in-line and non-destructively. This technology operates through two avenues: (ⅰ) morphological information, including cell types and cell status, which can be extracted through segmentation of cell images such as cell nucleus, single cells, and cell clusters [[100], [101], [102], [103], [104]]; and (ⅱ) non-morphological information, including the genomic, proteomic, and metabolic [[105], [106], [107], [108], [109], [110]], which can also be gleaned through cell images.

Moreover, the obtained in-line data is input into the CMA/CPP prediction model, offering a real-time assessment of the congruence between ongoing culture conditions and desired outcomes, which will be subsequently fed back to the control strategy for in-line correction. For instance, Yang Zhao's group [111] has employed AI algorithms to intervene in the differentiation process of pluripotent stem cells (PSCs) into cardiomyocytes (CMs). They optimized the initial state of PSC colonies and applied real-time, non-destructive characterization and regional purification of cells during the culture process. As a result, they significantly improved the efficiency of CM induction, increasing the successful differentiation rate from 63 % to 94.7 % (Fig. 5c).

Furthermore, inputting in-line data into a process prediction model can forecast the future status of cell culture, enabling proactive intervention to mitigate risks. Notably, Ming-Dar Tsai's group [112] has employed the RNN algorithm to predict the future status of cell colonies during the reprogramming process of human induced pluripotent stem cells (hiPSCs) based on time-lapse bright-field microscopy images.

### 3.4 Design of bioink materials

Owing to the tissue- and function-specific nature of the repaired part [113], personalized design for bioink materials is necessary to fulfill specific properties (or CQA) [[114], [115], [116], [117], [118]]. Typically, this entails significant domain expertise and extensive trial and error, which are both time-consuming and expensive. However, ML methods offer avenues for improvement in two key aspects: (ⅰ) reduction of time and cost associated with property characterization for high-throughput screening of bioink materials, and (ⅱ) modeling the intricate mapping relationship between the fingerprints and properties of bioink materials, thereby enabling the property prediction and expediting the design process.

**ML-based property characterization of bioink materials:** Rheological properties are paramount in characterizing bioink materials, yet traditional characterization methods relying on rheometers suffer from high cost and limited throughput. These challenges can be addressed through ML-based characterization methods. For instance, Min Zhang's group [119] has used characterization data from near-infrared (NIR) spectroscopy and low-field nuclear magnetic resonance (LF-NMR) as inputs of ML models, such as CNN, SVM, long short-term memory (LSTM), and Transformer, to predict the rheological characteristics of hydrogel inks. Blake N. Johnson's group [120] has devised a measurement approach utilizing robots and ML models, enabling high-throughput and cost-effective determination of gelation status. These approaches have offered promising solutions for rapid and high-throughput characterization of rheological properties. Additionally, Safa Jamali's group [54,121] and Gareth H. McKinley's group [122] have employed ML methods, specifically based on physics-informed neural networks (PINNs), to develop precise rheological constitutive models of hydrogels. Termed as “digital rheometer twins”, this approach can accurately predict the complete rheological behavior of hydrogels with minimal experiments (Fig. 5d), offering a potential alternative to physical rheometers and markedly reducing characterization-related time and costs. Furthermore, the rheological constitutive model potentially contributes to a deeper understanding of the rheological mechanism underlying bioink materials.

**ML-based design of bioink materials:** The properties of bioink materials encompass the form-based and function-based characteristics (Table 1). Bioink materials, represented by hydrogels, typically exhibit multi-scale structures [123]. Consequently, we attribute the construction of ML models to fingerprints at three scales:

(ⅰ) **Property-based scale:** This approach focuses primarily on composition ratios and rheological properties. Adjusting composition ratios of bioink materials is straightforward for the operator and enables rapid modification of various properties. Studies have utilized ML models to predict a range of properties, including printability [65,124], rheological properties [[124], [125], [126]], gelation properties [[127], [128], [129]], mechanical response [130], degradation properties [131,132], swelling properties [[133], [134], [135], [136]], and cell behavior [126,137,138], with composition ratios of bioink materials serving as inputs. Additionally, as the rheological properties of bioink materials serve as predictors of printability [37], studies employing ML models have explored the relationship between rheological properties and printability [72,125], enhancing the understanding of the printing mechanism. For instance, Jürgen Groll's group [72] has utilized interpretable ML methods to analyze the effect of rheological fingerprints on printability and the interaction between these fingerprints, further elucidating printing behavior. This offers an excellent reference utilizing ML models for risk assessment and mechanism analysis in QbD of 3D bioprinting (Fig. 5e). Besides, the mechanical response has also been used as inputs to predict degradation properties [131] and cell behavior [138]. Furthermore, ML models have been employed to investigate the fabrication process of novel bioinks such as microgel particles [139].
In recent years, the emerging 4D bioprinting has opened an exciting avenue for engineering functional tissues and organs [140]. Through the application of specific external physicochemical stimuli, such as the temperature, pH, ion concentration, electric field, and magnetic field, these 4D BPPs can undergo controlled shape morphing, facilitating the attainment of specific biological functions. This requires bioink materials to exhibit stimuli-responsive swelling properties. In this context, there have been studies using ML models to build the mapping relationship between external physicochemical stimuli (such as the pH, temperature [141], time [142], and external force constraints [143]) and swelling properties of hydrogels (such as the swelling ratio and drug release ratio).

(ⅱ) **Structure-based scale:** Analogous to the natural extracellular matrix (ECM) [144], bioink materials such as hydrogels possess microscale network structures. ML models have been utilized to predict various properties, including mechanical response [[145], [146], [147]], degradation properties [131], and swelling properties [148], with microstructures of bioink materials serving as inputs. For instance, Linxia Gu's group [146] has integrated finite element analysis with CNN models, employing microstructural images as input to predict the mechanical properties of collagen-based biomaterials.
(ⅲ) **Molecule-based scale:** Due to the challenges of conventional hydrogel-based bioinks in simultaneously meeting the requirements for both printability and biocompatibility, various reinforcement strategies have emerged to improve bioinks' properties [149]. For example, driven by supramolecular interactions, such as hydrogen bonding, hydrophobic interactions, and electrostatic interactions, peptides can self-assemble to form supramolecular hydrogels [150]. The diversity of molecular structures and the complexity of supramolecular interactions present significant challenges in designing self-assembling peptides. In this context, studies have emerged employing ML models to accelerate the discovery of self-assembling peptides [[150], [151], [152], [153]]. For instance, Junfeng Shi's group [150] has proposed the HydrogelFinder workflow, which utilizes molecular structures as inputs and gelation properties as outputs (Fig. 5f). This workflow has successfully identified nine novel self-assembling peptide hydrogels that had not been previously reported. To summarize, molecular-based modeling has demonstrated significant potential in mechanistically developing bioink materials with novel properties, particularly for supramolecular bioinks.

In contrast to the DoE paradigm, which relies on adjusting composition ratios, the ML-based data-driven paradigm can predict the macroscacle properties of bioink materials using micro-nanoscale fingerprints, such as network structures and molecular structures, achieving multi-scale modeling. Machine learning serves as a powerful tool for comprehending diverse behavioral mechanisms of bioink materials, including rheology, gelation, mechanical response, degradation, swelling, and cell behavior, as well as for designing bioink materials with specific or even potentially groundbreaking properties.

## 4 AI-driven approaches for model structure

**Fig. 6:**
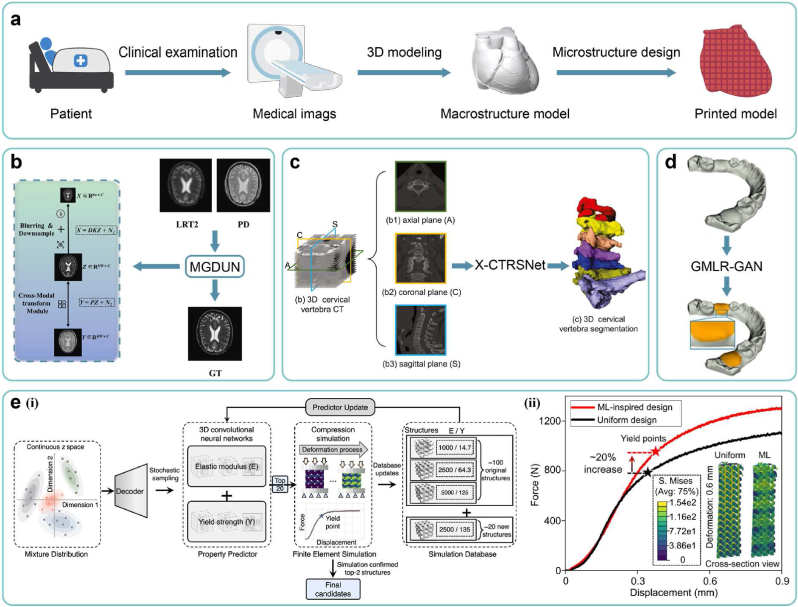
AI-driven approaches for model structure. (a) A pipeline of personalized design of printed models. (b) A schematic diagram of super resolution reconstruction based on multi-contrast MRI images. Copyright 2023, Elsevier. (c) A schematic diagram of organ segmentation and 3D reconstruction based on orthogonal CT images. Copyright 2022, Elsevier. (d) Experimental results of tooth gingival margin line reconstruction based on the adversarial learning method. Copyright 2022, Elsevier. (e) (ⅰ) A workflow of active learning loop for high-performance microstructure discovery with 3D-CNN, (ⅱ) Experimental displacement-force curves of the ML-inspired design versus uniform design. Copyright 2023, Nature Publishing Group.

Upon finalizing bioink formulations, another critical element is the design of the printed models' structure. Due to the tissue, structure, and function specificity of BPPs, structures of printed models necessitate personalized design to meet the property requirements. The typical process of designing printed models involves the following steps: first, acquiring medical images of the patient's target organs/tissues using imaging modalities such as CT and MRI, as described in Section 4.1; second, performing 3D modeling based on these medical images to generate the macrostructure model, as described in Section 4.2, 4.3; and finally, designing the internal microstructure, as described in Section 4.4. AI technology can be applied to each UO within these processes to expedite the precise design of printed models with personalized structures (Fig. 6a).

For the precise design of in vivo implants, we summarize multi-scale fingerprints of printed models (serve as CMA/CPP) critical to achieve various properties (serve as CQA) (Table 2). For example, at the macro scale, the external shape of the implant (such as the bone implant) should match the anatomical shape of the defect, which can improve the cosmetic effect and structural support; at the micro scale, the radius and shape of the implant's pores affect the transport properties, mechanical properties, and cell behavior responses; at the nano scale, the nano-topography affects cell behavior responses [13]. AI models aim to sense and optimize these fingerprints to enhance the overall performance of printed models. It is worth mentioning that this chapter focuses on the design of structural fingerprints, where Sections 1, 2, 3, 4 describe the design of the external macrostructure, and Section 4.4 describes the design of the internal microstructure. Additionally, the bioinks used for printed models discussed in this chapter include not only bioinks with cells (such as hydrogels) but also biomaterial inks without cells (such as thermoplastic polymers) [154].

**Table 2:** Multi-scale fingerprints of printed models.

Scale	Examples of fingerprints	Affected properties
Macro	Macro morphology, physicochemical properties of bioinks, external physicochemical stimuli	Transport, mechanical, biological
Micro	Fiber: orientation, size	Transport, mechanical, biological
	Pores: shape, size, distribution	
Nano	Surface pattern	Biological

### 4.1 Acquisition of high-resolution medical images

To acquire the macroscopic morphology of damaged organs/tissues in patients, medical imaging techniques such as CT, MRI, PET, and ultrasound (US) are indispensable. High-resolution medical images serve as the foundation for creating precise printed models. However, the resolution of conventional medical images, typically at the mm level, falls short of that of most 3D bioprinters, typically operating at the um level. Consequently, this limitation hinders 3D bioprinters from fully realizing their manufacturing potential. Moreover, factors such as patients' motion during the imaging process can lead to artifacts in certain areas of images, significantly diminishing the clarity. Despite ongoing advancements in higher-resolution medical imaging equipment [155], widespread clinical application remains challenging due to factors such as bulkiness, cost, and radiation exposure.

Super-resolution technology [156], employing the deep learning methodology, offers remedies to these challenges [45]. By utilizing algorithms such as CNNs [157], convolutional recurrent neural networks (CRNNs) [158], variational networks [159], and attention mechanisms, this technology generates high-resolution images (HRIs) from low-resolution images (LRIs). It has found application across various medical imaging modalities, such as CT [[160], [161], [162]], MRI [[163], [164], [165]], and PET [166] in diverse organs, such as the brain, liver, lung, and abdomen. However, single image super-resolution (SISR) suffers from limited vertical resolution due to the absence of inter-layer information. Additionally, single imaging modalities lack the ability to adequately sense organs with complex tissue distributions, thus compromising resolution. To address these shortcomings, super-resolution technologies utilizing multiple medical images as inputs have emerged. These approaches can enhance the resolution beyond that achievable by SISR alone. Depending on the forms of input images, we categorize these methods into two main categories:

(ⅰ) **Based on volumetric images:** Leveraging medical volumetric images allows for the consideration of hidden spatial relationships between image layers. Studies have employed AI algorithms such as 3D convolutional neural networks (3D-CNNs) and GANs to perform super-resolution on brain MR images [167] and abdominal CT images [168].
(ⅱ) **Based on multi-modal images:** In the field of MRI, multi-contrast super-resolution (MCSR) technology amalgamates information from multi-contrast images to produce high-resolution images with improved tissue contrast and reduced noise. This approach has been applied to enhance the resolution of MR images of tissues such as the brain and knee (Fig. 6b) [169].

### 4.2 3D modeling of target organs/tissues

Upon acquiring high-resolution medical images, the subsequent task involves identifying and segmenting the region of interest pertaining to the target organs/tissues within the images, followed by constructing a 3D model. AI-based methods for image segmentation and 3D reconstruction offer advantages over manual operations, mitigating subjective errors while providing rapidity and repeatability. Studies have demonstrated the efficacy of employing AI algorithms, such as 3D-CNNs, to segment and reconstruct serial medical images (such as CT and MR images) depicting various organs (such as the abdomen [170,171], liver [172], kidney [173], chest [174], and head [175]) and tissues (such as the vasculature [176], muscles [177], and tumors [[178], [179], [180]]).

However, single-perspective and single-modal imaging methodologies present several limitations. To enhance the precision of segmentation and 3D reconstruction, we summarize two main approaches commonly utilized:

(ⅰ) **Based on multi-perspective images:** AI algorithms can segment medical images from diverse perspectives, such as orthogonal CT and X-ray images. By voting or weighting the segmentation results, this approach can effectively reveal morphological features of target organs/tissues from multiple perspectives, which has been applied to various targets, including spines (Fig. 6c) [181] and liver tumors [182].
(ⅱ) **Based on multi-modal images:** Leveraging multi-contrast MR images (such as T1, T1ce, and T2) or cross-sensor images (such as CT, PET, and MRI) and employing MML methods allows for harnessing the strengths of various image types. Studies have successfully achieved segmentation and 3D reconstruction of organs/tissues with the above methods, such as the pancreas [183], breast tumors [184], and brain tumors [185].

Last but not least, existing studies mainly focus on specific performance metrics of AI models (such as ROC), with little attention paid to the regulatory and security aspects of models. In fact, the reproducibility of AI model training results is often poor due to variability stemming from multiple factors, including datasets, optimization processes, hyperparameter choices, model architecture, and hardware configurations. In the context of clinical deployment, the lack of transparency in the training process and the poor reproducibility of results present significant challenges for the regulation of AI models. To address these issues, the first step is to provide a standardized and detailed description of the model design and training process. Then, a robust evaluation system for model performance should be established. Finally, technical measures should be adopted to minimize variability from multiple sources [186].

### 4.3 Generation of implant models

Upon acquiring the 3D model of the damaged organ/tissue, the design of personalized implants necessitates consideration of the macrostructure to align with the site of damage. In the realm of cranial and tooth restoration, conventional virtual design methods, including mirroring technology, statistical shape models, and deformable templates, are operation-intricated and time-intensive, limited in the application of specific defect types [187]. In light of this, generative AI technology has emerged as a transformative solution for automating the generation of implant macrostructures based on any provided 3D model of damaged organs/tissues. This advancement supplants manual operations, significantly enhancing universality, rapidity, and reproducibility. In cranial restoration, researchers have applied AI algorithms such as GANs [188,189] and AEs [190] to fabricate personalized implants. Similarly, in tooth restoration, investigations have leveraged AI algorithms such as 3D-CNNs [191] and GANs [[192], [193], [194]] to engineer crucial structures such as the occlusal surface and gingival edge of compromised teeth (Fig. 6d).

### 4.4 Design of microstructures

Following the aforementioned steps, the macro external shape (or macrostructure) of the printed model is obtained, necessitating personalized fine design of its internal microstructure to fulfill property requirements [[195], [196], [197]]. Given the prohibitive cost of clinical trials, the DoE paradigm is not viable. Instead, the computational paradigm can simulate the transport and mechanical properties of the microstructure through numerical simulation methods such as CFD and FEA. However, the high complexity of the microscacle topological structure results in a high-dimensional parameter space, demanding substantial computational resources. By incorporating the ML-based data-driven paradigm, datasets can be constructed from simulation data. The trained ML model can replace numerical calculations, significantly reducing the computational burden.

In the realm of metamaterials, leveraging the two approaches of inverse design outlined in Section 2.2.2, studies have combined machine learning and numerical simulation to inversely design microstructures, further details can be found in other comprehensive reviews [198,199]. Given their multi-scale structures akin to human natural organs/tissues (such as the bone, cartilage, and skin) [[200], [201], [202]]*,* these theories and methods are emerging in the field of 3D bioprinting. Some studies first adopt ML approaches based on ANNs [[203], [204], [205], [206]] or CNNs [68,69,207,208] to establish forward design models, where the design parameters of microstructures (such as geometric parameters of unit cells) serve as inputs, and the resulting mechanical properties (such as the elastic modulus, stiffness, and yield strength) serve as outputs. These forward design models can replace finite element analysis and real experiments, enabling rapid prediction of candidate designs' feasibility. Subsequently, searching strategies (such as particle swarm optimization algorithms [204], genetic algorithms [203], Bayesian optimization [208], and active learning methods [68]) are integrated with the established forward design models to determine the optimal microstructure design with desired mechanical properties. For instance, Peng Wen's group [68] has employed 3D-CNN models as forward design models, with active learning as the search strategy, to design topologies of printed scaffolds' microstructures (Fig. 6e ⅰ). In animal experiments, this approach has ultimately achieved fixed elastic modulus and improved yield strength by 20 % compared to those through uniform design (Fig. 6e ⅱ). Moreover, other studies utilize ML approaches based on AEs [69] and ANNs [205,206] to establish inverse design models, where expected mechanical properties serve as inputs, and the optimal microstructure design serves as outputs.

Notably, the transport and mechanical properties impose conflicting requirements on the microstructure (such as the porosity for stiffness and transport properties), thereby machine learning integrated with multi-objective optimization is necessary for the optimal design of microstructures [204]. In addition, ML models predicting properties rapidly facilitate the discovery of microstructures with rare or extreme properties, which is difficult for traditional paradigms [209,210].

Additionally, the emergence of 4D printing has introduced significant challenges in microstructure design. In 4D bioprinting, the heterogeneous spatial distribution of stimuli-responsive bioinks dictates the unique shape-morphing behaviors of BPPs in response to external stimuli [211]. Given the complexity of these spatiotemporal dynamics, the conventional DoE paradigm faces significant limitations in efficiently and precisely designing the spatial distribution of bioinks to achieve the desired shape-morphing behaviors. Recently, in the field of 4D printing of active composites, studies [[211], [212], [213]] have employed ML methods to design the spatial distribution of ink materials. This offers valuable insights for the application of ML approaches in the 4D bioprinting field.

Last but not least, cell behavior is another critical consideration in microstructure design. Recent studies [214,215] have employed ML approaches to model the mapping relationships between microstructures (such as the fiber diameters/orientations and the pore size distributions) and cell behaviors (such as the cell numbers and cell morphologies). However, the datasets in these studies are derived from post-bioprinting experimental results, which are costly and time-consuming. Concerning this, a promising development direction is multi-scale modeling integrated with spatiotemporal models of cell behavior [216]. Ideally, in the R&D stage, it is necessary to fully predict the properties of the printed model at both the spatial scale (organ, tissue, and cell) and the temporal scale (dynamic evolution over time) for systematic optimization design. However, currently, the design of printed models mainly focuses on the non-biological properties at the organ/tissue scale (such as the transport and mechanical properties), with less attention paid to biological properties at the cell scale. By combining spatiotemporal models of cell behavior and machine learning, it is expected to model the relationship between the microenvironment and the dynamic response of cell behavior. Furthermore, by integrating them into numerical simulations, the multi-scale properties of printed models, including transport properties, mechanical properties and the evolution of cell behavior, can be fully predicted. In this regard, there have been preliminary attempts in relevant studies [217,218].

## 5 AI-driven approaches for printing process

Upon acquiring the printed model, the printing process element entails the precise manufacture of the designed multi-scale structure while ensuring cell viability. To enhance printing quality, the optimal printing parameters should be designed off-line before the formal printing process, as described in Section 5.1. Subsequently, during the formal printing process, the printing parameters need to be adjusted in real time to maintain control over printing quality, as described in Section 5.2.

### 5.1 Design of printing parameters

According to the QbD, the bioink parameters (serving as CMA) and printing parameters (serving as CPP) collectively determine the printing quality (serving as CQA), primarily focusing on printability and cell viability. Traditional paradigms have encountered the dilemma of “precision-cost”. In contrast, the ML-based data-driven paradigm can model mapping relationships between CMA/CPP and CQA at a manageable cost, facilitating rapid determination of the optimal design space to enhance printing quality.

**Fig. 7:**
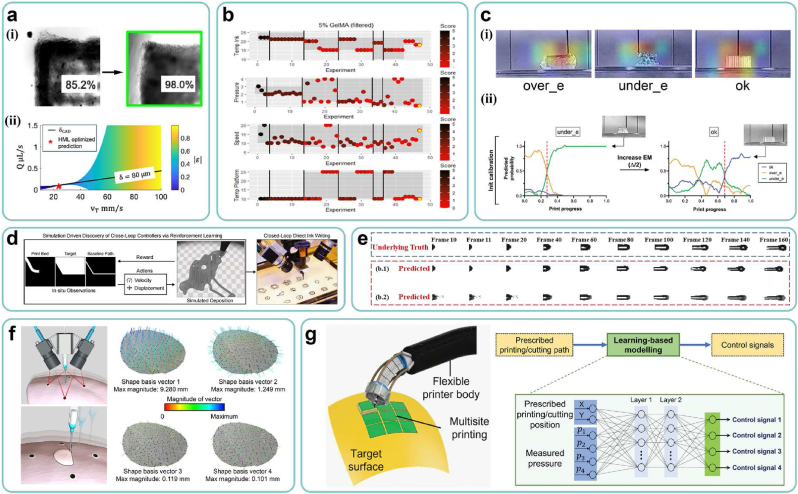
AI-driven approaches for printing process. (a) (ⅰ) Experimental results of printability improvement through machine learning optimization, (ⅱ) Phase diagrams of the design space for printing parameters. Adapted with permission from J.M. Bone, C.M. Childs, A. Menon, B. Póczos, A.W. Feinberg, P.R. LeDuc, N.R. Washburn, Hierarchical Machine Learning for High-Fidelity 3D Printed Biopolymers, ACS BIOMATER SCI ENG, 6 (2020) 7021–7031. Copyright 2020, American Chemical Society. (b) Optimization process of printing parameters via Bayesian methods. Copyright 2021, Elsevier. (c) (ⅰ) Prediction results of CNN models on the printing status. (ⅱ) Experimental results on automatic optimization of printing parameters. Copyright 2022, AccScience Publishing. (d) The schematic diagram of a closed-loop control strategy via reinforcement learning. Copyright 2022, ACM. (e) Prediction results on the evolution process of inkjet printing. Copyright 2023, Elsevier. (f) Sensing results of surface deformation using PCA algorithms. Copyright 2020, AAAS. (g) A schematic diagram of the printing head's motion controllers, built by ANN models. Copyright 2023, Wiley.

Within the framework of Section 2.2.2, Table 3 provides a summary of examples in which AI technology has been employed for process modeling and parameter optimization across various 3D bioprinting processes. Different types of processes emphasize various aspects of CQA, CMA, and CPP, as documented in prior research [[219], [220], [221], [222], [223]]. For instance, in photocuring printing, digital masks serve as generalized CPP. Shaochen Chen's group [224,225] has employed deep learning methods to optimize the design of digital masks, mitigating the impact of cell scattering on printability. The trained ML models can be used to determine the design space and optimal combinations of printing parameters. For instance, Newell R. Washburn's group [226] has employed the hierarchical machine learning approach to optimize printing parameters, improving printability from 85.2 % to 98 % (Fig. 7a ⅰ). Furthermore, through the trained ML model, phase diagrams of the design space for printing parameters are generated, visually illustrating the distribution of printability (Fig. 7a ⅱ). In addition, Bayesian methods have been utilized as active learning strategies to optimize printing parameters [[227], [228], [229], [230]]. For instance, Gordon Wallace's group [228] has employed Bayesian optimization to guide experimenters in adjusting printing parameters. As the number of experimental iterations increases, printability continuously improves until the optimal combination of printing parameters is found (Fig. 7b).

**Table 3:** Examples of AI applications for process modeling and parameter optimization in 3D bioprinting.

Process category	CMA	CPP	CQA	AI Model	Ref
EBB	C_ink_	Q, V_T_, D_nozzle_	Shape fidelity	Hierarchical machine learning (HML)	[226]
	η, $G^{\prime }$	$\Delta P,D_{n},V_{n}\text{,}$$L_{n}$	Printing resolution	Rheology-informed hierarchical machine learning (RIHML)	[240]
	GelMA composition	Ink reservoir temperature, pressure, speed, platform temperature	Filament morphology, layer stacking	Bayesian optimization	[228]
	–	Air pressure, biomaterial ink temperature, print speed	Print resolution	Bayesian optimization	[227]
	FSA concentration	Nozzle size, printing temperature, pneumatic pressure	Printability	Gaussian process regression (GPR)	[241]
	–	Rb, Rs, Lu, Ll, Rm (nozzle geometrical parameters)	Maximum shear stress	Gaussian process (GP)	[242]
	Material composition	Printing speed, printing pressure, scaffold layer, programmed fiber spacing	Printing quality	RF	[243]
	Biomaterial concentration	Nozzle temperature, printing path height	Printability	SVM	[244]
	Material concentration, solvent usage	Crosslinking mechanism and duration, printer settings, observation duration	Cell viability, filament diameter, extrusion pressure	Support vector regression (SVR), linear regression (LR), random forest regression (RFR), RF, logistic regression classification, SVM	[245]
	Gelatin concentration	Printing speed, flow rate, temperature	Printability, Precision	Fuzzy inference system (FIS)	[246]
	Dilution percentage of bioink	Nozzle pressure, printing speed,	Line width	Fuzzy inference system (FIS)	[247]
	Viscosity, growth factor concentration	Gauge pressure, build orientation, printing speeds	Print resolution	LSTM	[248]
	–	Printing speed, pressure of extrusion, infill percentage	Gel weight, surface area, topographical heterogeneity	SVM, Gaussian model	[249]
	Biomaterial type, biomaterial concentration, crosslinker concentration, cell type, cell number	Crosslinking time, printing pressure, movement speed, nozzle size, cartridge temperature, bed temperature	Cell viability	Bayesian optimization, ANN	[229]
	Material's weight fraction	Extrusion pressure, print speed, z-height	Filament width	Linear regression	[250]
	–	Nozzle temperature, infill density, layer height, printing speed	Tensile strength	Linear regression, RFR, XGB regressor, LGBM regressor, ANN	[251]
	–	Air pressure, biomaterial ink temperature, print speed	The width of printed filament	Bayesian optimization	[227]
	Material's weight fraction	Extrusion pressure, print speed, nozzle diameter, z-height	Filament width	Linear regression	[250]
	–	Layer height, nozzle travel speed, and dispensing pressure	Time, porosity, and geometry precisions	Multi-objective Bayesian Optimization	[252]
	–	Printing speed, extrusion pressure	Width average, width variance height average and height variance	SVM	[253]
	–	Nozzle tip to collector distance	Jet radius profile	GP	[230]
	–	Ratio of the collector speed over the jet speed at the point of interest	Lag distance		
	Cell type	Wall shear stress, exposure time	Cell viability	Multi-layer Perceptron (MLP)	[254]
DBB	Viscosity, surface tension	Voltage, diameter of the nozzle	Droplet formation	MLP	[255]
	Viscosity, surface tension	Voltage, nozzle diameter	Droplet deformation	Fully connected neural network (FCNN)	[256]
	Polymer concentration	Voltage, dwell time, rise time	Droplet velocity and volume	Ensemble learning	[257]
	–	Standoff height, applied voltage, ink flow rate	Droplet diameter	Regression analysis (RA), backpropagation neural network (BPNN), neural network trained with genetic algorithm (GA-NN)	[258]
	The type and concentration of solute and solvent	Inner diameter (D_in_), outer diameter (D_out_), the materials of the nozzle and grounded substrate, volumetric flow rate (Q), the distance (L) between needle and grounded substrate, the environmental gas, the applied voltage (V) between the ground electrode and needle	Spraying patterns	ANN,SVM	[259]
	Viscosity (μ), Density (ρ), Conductivity (K), Surface tension (γ), Relative permittivity (κ)	Nozzle internal diameter (Din), nozzle external diameter (D_out_), distance between nozzle and grounding electrode (L), applied voltage (V), flow rate (Q)	Droplet diameter	ANN	[260]
	Dimensionless number Z	Rise time, drive'voltage, dwell time, fall time	Drop velocity, drop formation	SVM, KNN, RFs, extreme gradient boosting (XGBoost), MLP	[261]
	Bioink viscosity, cell concentration	Nozzle size, printing time, printing pressure	Droplet size	DT, RF, PageRank, MLP, LSTM	[262]
LBB	–	Digital mask	Printing fidelity	U-Net-like neural network	[224,225]
	–	Digital mask	Printing fidelity	3D U-Net	[263]
	–	Digital mask	Printing fidelity	Convolutional Auto-Encoder (CAE)	[264]
	–	Digital mask	Printing fidelity	Deep neural networks	[265]
	GelMA concentration	UV intensity, UV exposure time, layer thickness	Cell viability	Ensemble learning model	[266]
	–	Exposure time, light intensity, print speed, laser current, laser power, infill density	Young's modulus	ANN	[232]
	Resin viscosity	Cross section size used for synthetic dataset construction, manufacturing velocity, PDMS thickness, constrained surface type, duration of frame, video projection time, groove width, groove depth, cross section size used for separation force boundary construction	Printing success or failure, optimum printing speed	KNN, SVM, decision tree, logistic regression, quadratic discriminant analysis, GP, naiveBayes, ANN, ensemble learning model, Siamese network	[267]

However, most existing studies are applicable only to single and straightforward working scenarios. As the construction of natural heterogeneous tissues necessitates increasingly complex bioinks and printed models, it is crucial for ML models to address such complexities. We identify two example aspects of these complexities:

(ⅰ) **Multiple components:** Multi-component bioinks have introduced tremendous diversity to bioink systems. However, existing ML models typically employ composition ratios as input, restricting their applications to specific bioink systems and lacking in universality. Essentially, ML models represent potential mappings between inputs and outputs. Therefore, for bioink systems sharing similar printing mechanisms, such as GELMA-based and HAMA-based used in the direct ink writing process, a universal ML model is theoretically feasible. In this regard, a critical step is the extraction of mechanism-level features that directly influence printing behavior as inputs, such as the rheological curves. Following training, for various bioinks, the single universal ML model can yield satisfactory prediction results with minimal experiments through fine-tuning of transfer learning methods.
(ⅱ) **Gradient structures:** Naturally, heterogeneous tissues exhibit a zonal gradient structure, requiring dynamic adjustment of printing parameters over a large range in response to gradient changes of structures and properties [231]. Consequently, ML models must possess high prediction accuracy within a broader design space of printing parameters. A relevant study has been conducted in this regard [232].

Although ML methods have made progress in optimizing printing parameters, the process of manually constructing datasets is time-consuming. To expedite the search for optimal printing parameters, an increasingly promising approach involves constructing a fully automatic and universally applicable search procedure, offering significant practical utility. In this approach, the 3D bioprinter autonomously performs local printing tests for any input bioink and printed model, evaluates the printing quality, and adjusts the printing parameters to determine the optimal settings. Notably, the ML model transcends its conventional role of modeling mapping relationships between inputs and outputs; it also assesses printing quality and serves as the adjustment strategy of printing parameters. For instance, Jianhua Zhou's group [233] has automatically traversed three printing parameters in a special-designed 3D bioprinter, employing a CNN-based algorithm to classify printing quality into three categories and derived a process phase diagram to determine the optimal design space of printing parameters. Although this study achieved high-throughput screening of printing parameters, the process was partially automated. Carmelo De Maria's group [234] has employed a CNN-based algorithm to classify printing quality into three categories and iteratively adjusted printing parameters using a dichotomy-like strategy to obtain the optimal settings (Fig. 7c ⅱ). This process was fully automated but did not optimize all printing parameters. Filippos Tourlomousis' group [230] has employed Bayesian optimization and active learning to automatically adjust the ratio of the collector speed over the jet speed in the MEW process, aiming to achieve the minimum Lag distance.

In the broader field of 3D printing, studies [73,75,[235], [236], [237], [238]] have embraced the concept of closed-loop iteration by employing ML methods to automatically search for the optimal printing parameters. Analogously, such methodologies hold promise in the domain of 3D bioprinting. A critical consideration in this context is the selection of printability metrics. During the process of searching for optimal printing parameters prior to formal printing, it is judicious to print simple and standard test models rather than complex and complete input models. Therefore, it becomes imperative to swiftly identify printability metrics [37,239] (such as corners, filament diameters, and layer spacing) that may influence the final printing quality [238] and select test models accordingly, necessitating a comprehensive understanding of the printing process.

### 5.2 Control of printing processes

Owing to the inevitable occurrence of process drift and model error, deviations in the printing quality from the anticipated standards may arise within the actual printing process, if the optimal printing parameters derived from off-line design is continuously employed [10]. To maintain consistent control over printing quality, the implementation of in situ monitoring and in-line correction becomes imperative.

Within the framework of Section 2.2.3, Table 4 provides a summary of examples, where AI technology integrated into diverse sensors has been employed for in situ monitoring and in-line correction across various 3D bioprinting processes. Among them, the combination of cameras and CNN-based algorithms is the most common technical solution [234,[268], [269], [270]]. Some studies [271,272] have utilized reinforcement learning methods to ascertain adjustment strategies for printing parameters, enabling adaptation to the dynamic environment (Fig. 7d). In addition, other studies [273,274] have leveraged deep learning methods to predict the entire droplet evolution process in inkjet printing, enhancing the understanding of printing behavior and facilitating early defect detection (Fig. 7e).

**Table 4:** Examples of AI applications for in situ monitoring and in-line correction in 3D bioprinting.

Process category	Sensor	Input	Output	AI Model	Type of AI Model	Ref
EBB	Camera	Video frame	Printing status	CNN	CQA prediction model	[234]
	Camera	Video frame	Extrusion status	LSTM autoencoder	CQA prediction model	[302]
	Camera	Image	Printing anomaly	CNN	CQA prediction model	[269]
	Camera	Video frame	Velocity of the printing head, offset from the baseline printing path	Reinforcement learning	Control Strategy	[271]
	Infrared thermocouples	Three features extracted from the raw sensor signals	Printing status strand width, strand height, strand fusion severity	KNN, SVM, RF, ANN	CQA prediction model	[303]
		Material temperature, extrusion pressure, print speed, the location in the strand	Regime classification, width prediction, height prediction			
	Stereo cameras	Surface shape data	Shape basis vectors	PCA	CPP prediction model	[292]
	Camera, pressure sensor	Time-varying 2D printing head position (X, Y), SMAMs pressure (p_1_, p_2_, p_3_, p_4_)	The displacement of syringe plungers (l_1_, l_2_, l_3_, l_4_)	ANN	Control Strategy	[297]
DBB	Camera	Video frame	Droplet evolution in the printing process	Deep recurrent neural network (DRNN)	Process prediction model	[273]
	Camera	Video frame	Droplet evolution in the printing process	Network of tensor time series (TTS)	Process prediction model	[274]
	Camera	Video frame	Jetting status	MovileNetV2	CQA prediction model	[270]
	Camera	Droplet velocity at two different points	Cell count	LR, SVR, decision tree regressor (DTR), RFR, extra tree regression (ETR)	CQA prediction model	[304]
	Camera	Droplet size, aspect ratio, droplet velocity, satellite droplet	Droplet mode	Backpropagation neural network (BPNN)	CQA prediction model	[305]
	FPGA module for self-sensing signals acquisition	Two features extracted from the raw sensor signals	Nozzle jetting status	SVM, ANN, Gaussian naïve Bayes model	CQA prediction model	[306]
LBB	Camera	Video frame	Printing status	CNN-LSTM	CQA prediction model	[268]
	–	Digital Mask	Digital Mask	Reinforcement learning	Control Strategy	[272]

Currently, various imaging sensors, such as visible light cameras [234,268,270], laser displacement scanners [275], and optical coherence tomography (OCT) devices [[276], [277], [278], [279]], are utilized for in situ monitoring of the printing process. While they share common attributes such as non-destructiveness, low latency, and ease of integration, each possesses distinct advantages and drawbacks. Visible light cameras are prevalent due to their affordability and wide field of view; however, they can only capture 2D external profile information with limited resolution. Laser displacement scanners enable 3D profile imaging but lack the ability to penetrate the surface for internal feature extraction. OCT, which possesses certain penetration capabilities, can detect internal defects such as air bubbles but is characterized by high cost and limited field of view. Combining multiple imaging modalities can enhance the prediction accuracy and robustness of CMA/CPP and CQA/process prediction models. Within the broader realm of 3D printing, there have been studies [50,280] using MML methods to monitor the printing process in situ, combining several types of sensor images, which has important reference significance for 3D bioprinting. Furthermore, while the predominant emphasis remains on printability, it is imperative to underscore the significance of cell viability, especially for the sustained printing of organs/tissues with clinical volumes over extended durations. In this regard, several studies suggested sensing methods for in situ monitoring of cell viability [281,282].

In instances where severe defects are detected during the printing process, discarding the printed part leads to substantial waste, particularly concerning personalized small-batch BPPs. Therefore, compared with detecting the emerged defects, it is more meaningful to provide early warning of possible defects and intervene preemptively to maintain errors within acceptable limits. Analyzing the source of process drift is pivotal for achieving early defect warnings [283], encompassing environment-related (such as temperature fluctuations and mechanical vibrations), material-related (such as rheological nonlinearity of bioinks), system-related (such as motion errors in the driving mechanism) factors [17]. Setting up sensors to detect CMA/CPP related to process drift and collecting in-line data facilitate early defect warnings. ML models such as RNN, LSTM, gated recurrent unit (GRU), and Transformer excel in analyzing sequential information, enabling the prediction of future printing quality by CQA/process prediction models [284]. Moreover, combining off-line and in-line data can enhance prediction accuracy [285]. While these theories and methods are nascent in the realm of 3D bioprinting, relevant studies in 3D printing underscore the importance of similar methods [[284], [285], [286]].

Due to the limitations of traditional in vitro bioprinting [7], the emerging trend of in situ bioprinting technology, which directly prints at the patient's defect site, presents significant potential for clinical application. In contrast to traditional 3D bioprinting operating on static and typically planar workbenches, in situ bioprinting typically operates on dynamic and often curved biological surfaces within the body, such as the breathing lung or beating heart [17]. In this regard, traditional 3D bioprinters face notable challenges in two key aspects:

(ⅰ) **Degrees of freedom (DOFs):** The 3-DOF XYZ translational motion of traditional 3D bioprinters is difficult to match the 6-DOF surface.
(ⅱ) **Path planning strategy:** The strategy of planning the printing path in the pre-bioprinting stage struggles to adapt to the surface deformation caused by shrinkage, expansion and bending. This can lead to potential collisions between the printing head and the patient's body, resulting in printing failure or additional damage.

In addressing these challenges, in situ bioprinting technology leveraging robotics and AI offers promising solutions. Surgical robots, renowned for their high DOFs and precise control, have found widespread use in clinical surgery. Integrating printing heads with the ends of surgical robots presents a viable approach for in situ bioprinting [17]. Additionally, considering the high cost of surgical robots, some studies have explored the integration of industrial robots with printing heads [[287], [288], [289], [290], [291]]. Furthermore, employing closed-loop control methods based on predictive AI technology allows for precise real-time planning of the printing path. We present this employment in two aspects:

(ⅰ) **Sensing and prediction of dynamic environment:** Initially, 3D reconstruction is conducted based on visual features of real-time images to acquire the current printing surface morphology. A prior study utilized the PCA algorithm to extract key morphological features from the lattice information of pigs' lung surfaces marked by stereo cameras, facilitating rapid modeling of surface deformation during in situ bioprinting processes (Fig. 7f) [292]. Subsequently, the prediction of future surface morphology can be achieved based on historical surface morphology. Studies have employed AI algorithms such as LSTM [293,294], attention mechanisms [295], and DenseNet [296] to analyze sequential medical images, enabling tracking and predicting the motion of the 3D surface of target organs/tissues (such as the tumor and abdominal cavity). This approach holds significant potential for predicting complex deformation patterns of organs/tissues during in situ bioprinting processes.
(ⅱ) **Optimization of closed-loop control strategy:** Upon achieving 3D reconstruction of the printing surface, AI technology facilitates segmentation of the printing head and analysis of its spatial position, aiding in the correction of the printing path. In addition, AI technology can be used to construct control strategies and enable real-time correction of the printing head's position and posture, such as utilizing ANN models to build motion controllers (Fig. 7g) [297]. Reinforcement learning methods have also been employed to automatically plan and adjust the path of surgical robots, enhancing movement precision, efficiency, and adaptability to complex environments [[298], [299], [300], [301]]. These advancements hold significant reference value for in situ bioprinting based on surgical robots.

## 6 AI-driven approaches for function regulation

Upon completing the printing of high-quality structures, the final element is the function regulation of the printed structures. Primarily, the design of maturation conditions is imperative to functionalize the printed structures, thereby transforming them into BPPs with the requisite biological functions, as described in Section 6.1. Subsequently, for functionalized in vitro models and in vivo implants, their biological functions are characterized and assessed with non-destructive detection methods, facilitating applications such as drug screening, pathological/pharmacological studies, and assessments of clinical functions, as described in Section 6.2.

### 6.1 Design of maturation conditions

**Fig. 8:**
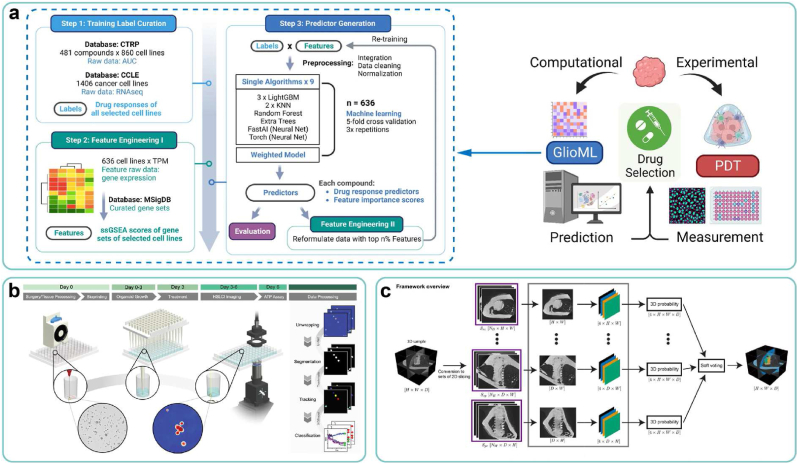
AI-driven approaches for function regulation. (a) A flow chart of the GlioML workflow. Copyright 2024, Nature Publishing Group. (b) A workflow of in situ high-throughput characterization of organoids (segmentation, tracking, and classification). Copyright 2023, Nature Publishing Group. (c) A schematic diagram of biodegradable bone implants segmentation based on SRμCT images. Copyright 2021, Nature Publishing Group.

Within bioreactors, specific external physicochemical stimuli (serving as CMA/CPP), including mechanical, electrical, photo, ultrasound, and soluble factors, are employed to modulate the biochemical and mechanical clues (or cell microenvironment) within BPPs [307]. And the cell behavior (serving as CQA) of BPPs is further regulated, such as proliferation, differentiation, and adhesion, to achieve the desired biological functions. Currently, the design of maturation conditions primarily relies on the DoE paradigm, which lacks quantitative theories and models. Recent studies have explored the utilization of the ML-based data-driven paradigm to model the mapping relationships between external physicochemical stimuli (such as biochemical stimulus [308], and drug stimulation [309]) and cell behavior (such as mechanobiological states [308] and drug responses [309]). For example, Yu Yao's group [309] has developed the GlioML workflow, incorporating nine ML models and a weighted ensemble model to predict the treatment response of glioma under different microenvironment characteristics, successfully identifying promising compounds and drugs for glioma treatment (Fig. 8a).

### 6.2 Characterization and assessment of functions

#### 6.2.1 In vitro models

AI-based sensing technology enables non-destructive characterization of the biological properties (serving as CQA) of in vitro drug/pathological models across multiple scales for drug screening and pathology/pharmacology studies. At the cell scale, studies have performed 3D segmentation and classification of cells/nuclei in images (such as CLSM [47,310] and bright-field microscopy [311]) to evaluate biological properties such as the cell shape and distribution. In addition, some studies have utilized spectral data (such as Raman spectroscopy [312,313], dielectric spectroscopy [314], and dielectric impedance spectroscopy [315]) to assess biological properties including the cell type, shape, distribution, and density. At the organoid scale, studies have conducted 3D segmentation and classification of single organoids in images (such as OCT [316], high-speed live cell interferometry [317], and bright-field microscopy [318,319]) to extract morphological features such as the shape and volume (Fig. 8b)**.**

#### 6.2.2 In vivo implants

Prior to the implantation of the BPP into the patient's body, it is imperative to assess whether the properties of the BPP and the health status of the patient (serving as CMA/CPP) are conducive to surgical implantation (serving as CQA). In the field of organ transplantation, studies have utilized ML models (such as decision trees [320], RFs [321], and ANNs [322,323]) to predict postoperative survival rates based on the healthcare data of donors and recipients. These findings hold significant reference value for the preoperative assessment of BPP implantation suitability.

Following implanting the BPP into the patient's body, monitoring the regeneration status of the damaged organs/tissues (serving as CQA) becomes essential using non-destructive detection methods. Several studies have employed non-destructive medical imaging techniques for continuous monitoring of implant regeneration status. For example, Julian Moosmann's group has employed the U-net algorithm to segment micro-computed tomography (μCT) images, enabling the precise characterization of the morphology of degradable bone implants and facilitating the time-resolved, quantitative assessment of their degradation efficiency (Fig. 8c) [324]. Daniel G. Anderson's group has used a clustering method to track the spatiotemporal morphology of implants encapsulating pancreatic islet cells in MR images, enabling the characterization of quantitative internal oxygen content [325].

In addition to facilitating regeneration of the damaged organs/tissues, implants themselves can function as biosensors for monitoring patients' health status [326]. Smart dressings applied to human wounds can utilize AI technology to sense the health status of the wound (such as the pH, temperature, humidity, and secretion concentration), which can evaluate and monitor wound regeneration status, as demonstrated in several studies [[327], [328], [329]].

## 7 Future directions

### 7.1 Construction of natural organs

#### 7.1.1 Non-destructive and rapid construction of digital twin organs

Natural organs exhibit intricate multi-scale heterogeneous structures, while patient-specific digital twin organs aim to capture this multi-scale information to construct transplantable replacements for natural organs [76]. This entails information at organ scale (such as 3D macrostructures), tissue scale (such as microstructures), and cell scale (such as cell types, spatial arrangement, and microenvironment). Currently, non-destructive imaging technologies at the organ scale, such as CT, MRI, and US, are relatively advanced. However, obtaining non-destructive 3D multi-scale models containing information at the tissue and cell scale remains very challenging, for which relevant studies have made preliminary attempts. For instance, the Human BioMolecular Atlas Program (HuBMAP) aims to create a multi-scale spatial atlas of the healthy human body at single-cell resolution [330]. It has already developed spatial atlases of organs/tissues, including the intestine [331], kidney [332], and placenta interface [333]. Techniques such as serial tissue sectioning [46] and optical tissue clearing [334,335] can extend the scale in the depth direction, enabling the acquisition of macro spatial information. These methods hold promise for constructing high-resolution 3D models of large-scale tissues. However, it's worth noting that these approaches rely on destructive sampling of in vitro organs/tissues, rendering them impractical for application in living patients.

Generative AI technology offers a promising solution for the non-destructive and rapid construction of digital twin organs. Multi-scale 3D models derived from in vitro organs/tissues can serve as the dataset to train the generative AI model, enabling it to learn spatial correspondence across different scales. Utilizing the patient's macrostructure model derived from non-destructive imaging as input, the trained AI model automatically populates the information at the tissue and cell scales that conforms to human anatomical principles, generating a digital twin organ enriched with multi-scale information. This approach circumvents the need for destructive sampling of patients' organs/tissues and lays the foundation for designing printed models of natural organ replacements. For instance, there have been relevant studies using AI methods to automatically generate hierarchical 3D vascular networks within organ-scale macrostructure models [[336], [337], [338]].

Another important issue to consider is the scale limitations of biomimicry. The traditional forward-design approach strives to replicate the structure of natural organs as precisely as possible. However, some studies [9] suggest that biomimicry may reach a limit where increased complexity no longer enhances functional outcomes. Additionally, technical and cost constraints render endless biomimicry of natural organs impractical, especially at the micro-nano scale. Considering clinical translation, the inverse-design approach aims to prioritize the regeneration of specific functions and the feasibility of manufacturing rather than emphasizing structural biomimicry. A potential strategy is to strike a balance between these two approaches by employing appropriate AI models at different scales. At the macro scale, the forward-design approach is applied, with AI models focusing on mimicking the macrostructures of natural organs. At the micro-nano scale, the inverse-design approach is used, with AI models prioritizing the enhancement of specific functions. The generated micro-nano structures are artificially designed rather than imitating natural organs, such as triply periodic minimal surfaces (TPMS). In this way, we can construct manufacturable digital twin organs.

#### 7.1.2 Multi-material 3D bioprinting of high-content printed models

**Fig. 9:**
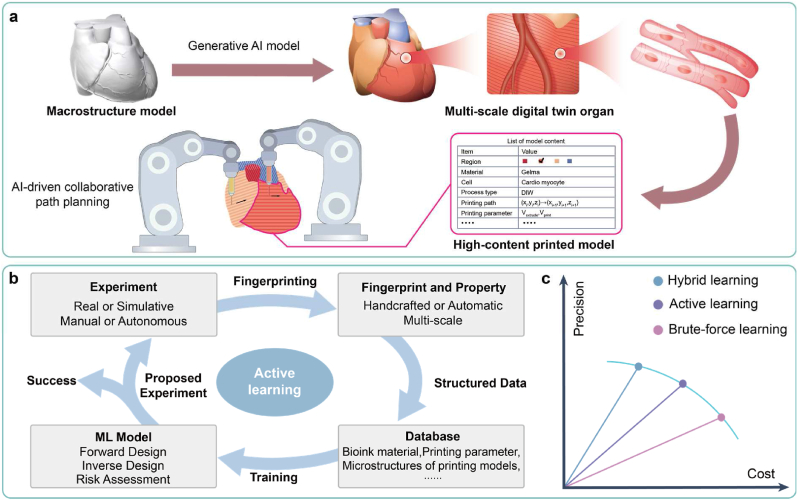
Future directions of AI technology in 3D bioprinting. (a) A pipeline of constructing natural organs. (b) A closed-loop active learning pipeline. (c) A “precision-cost” landscape of the brute-force learning, active learning, and hybrid learning.

Subsequently, the obtained digital twin organs should be converted into printed models that 3D bioprinters can interpret. Given the complexity of natural heterogeneous organs/tissues, multi-material 3D bioprinting, involving various bioinks, cells, and even printing processes, becomes one of the most promising construction techniques [18,339]. Currently, hybrid bioprinters integrating multiple bioinks and processing techniques are emerging [340,341]. In this scenario, the obtained multi-scale information of the digital twin organ needs to be further dissected into a high-content printed model, encompassing details such as the bioink material, cell type, process type, printing path, and printing parameter to guide subsequent multi-material printing (Fig. 9a).

Meanwhile, the prolonged printing cycle due to the organ scale of printed models poses risks such as cell sedimentation and contamination. To enhance manufacturing efficiency, a promising approach involves utilizing collaborative printing heads that independently deposit various bioinks simultaneously across distinct regions [291,340,342] (Fig. 9a). This collaboration of multiple printing heads introduces significant challenges for path planning, such as avoiding path interference. In the field of collaborative robots, studies have employed AI approaches to optimize path design for multi-robot arm collaboration, such as large language models, reinforcement learning, and computer vision [[343], [344], [345]]. These methods aim to enhance communication between robotic arms, ensuring interference-free operation while identifying more efficient paths, thereby significantly improving manufacturing efficiency.

### 7.2 Active learning and hybrid learning

Within the realm of 3D bioprinting, the absence of publicly available datasets poses a significant challenge for data-driven design. Consequently, the predominant cost of designing tasks stems from the construction of datasets, typically undertaken by researchers themselves through manual experiments or numerical simulations. In scenarios where datasets can be constructed with high throughput and low cost, conventional brute-force learning approaches (or learning from scratch) can be employed [55]. Initially, the dataset is constructed completely, and the ML model serves solely as a backend tool for automatic data analysis [43], aiding in comprehending underlying mechanisms. However, in scenarios where datasets have to be constructed with low throughput or high cost, the aforementioned methods prove impractical in terms of time or financial burdens.

One promising methodology is active learning [57,[346], [347], [348], [349]]. It can adaptively sample within the high-property region of the parameter space, which is typically the design space-related region of interest, while avoiding inefficient sampling in the low-property region [347]. The closed-loop active learning pipeline is illustrated in Fig. 9b. Initially, a set of preliminary experiments are conducted, and the built dataset is utilized to train the ML model. Subsequently, the trained ML model generates predictions across the parameter space, based on which the next experiment (or sampling point) is selected. The newly conducted experiment is then incorporated into the dataset, fostering a dynamic feedback loop aimed at iteratively enhancing the ML model's precision. Ultimately, once the ML model's precision aligns with the specified requirements, the training process concludes.

The selection of subsequent experiments holds paramount importance. Bayesian methodology, exemplified by Gaussian process (GP) models, can quantify prediction uncertainty. This capability facilitates the delicate balance between exploration and exploitation [57,346]. During the initial stage of active learning, experiments targeting regions of uncertainty are conducted to comprehensively explore the global parameter space. This process aids in discerning the distribution of properties and refining the ML model—a practice termed as exploration. As the ML model's precision improves and uncertainty diminishes, the focus gradually shifts toward conducting experiments aimed at achieving high-property predictions—a practice termed as exploitation. Through this approach, the ML model can efficiently identify high-property regions at a reduced cost and with heightened precision. While the active learning methodology has found widespread applications in the field of materials science [57,[346], [347], [348], [349]], its integration into 3D bioprinting is emerging [68,127,132,152,[227], [228], [229], [230],^261^].

Compared to brute-force learning, active learning effectively reduces costs through adaptive sampling but remains grounded in data and statistics. Evolving directions of the data-driven paradigm aim to further diminish cost and enhance precision, leading to the development of the hybrid-driven paradigm (Fig. 9c) [55]. The hybrid-driven paradigm amalgamates prior knowledge with experimental data through hybrid learning, utilizing prior knowledge to impose constraints on the functional space of ML models to accelerate the search process, facilitating modeling with small datasets, consequently reducing cost further [55].

Based on the forms of prior knowledge, the hybrid-driven paradigm can be divided into the following three categories:

(ⅰ) **Transfer learning:** This approach fine-tunes the pre-trained ML models rather than training them from scratch, enabling high precision with minimal experimental data [350,351]. For instance, Jennifer M Bone [352] has employed the transfer learning method based on hierarchical machine learning (HML) to optimize bioinks and support media in embedded bioprinting.
(ⅱ) **Multi-fidelity learning:** Combining high-cost/high-fidelity data from the DoE paradigm with low-cost/low-fidelity data from the theoretical, computational, and data-driven paradigms for training ML models can effectively address the lack of high-fidelity datasets [57,346]. For instance, Safa Jamali's group [54] has employed the multi-fidelity learning method to train PINNs to construct rheological constitutive models of hydrogels with a limited amount of experimental data.
(ⅲ) **Learning integrated with domain knowledge:** By incorporating domain knowledge such as expert experience [353,354] and physical laws [355] to constrain the functional space, ML models can achieve higher precision with minimal experimental data. For instance, Salil Desai's group [248] has integrated the physics model into the LSTM algorithm to predict printing resolution.

In summary, the application of the hybrid-drive paradigm in the field of 3D bioprinting is still in its nascent stages but holds significant potential for advancement.

### 7.3 Integrated automation of entire processes

In data-driven design, the quantity and quality of samples in the dataset are critical for ensuring the precision of the ML model. Conventional manual sampling methods are inefficient and prone to data noise. Currently, across various fields, such as materials science and chemistry, endeavors have been made to integrate AI technology with automation equipment, such as robots, to supplant manual sampling. This integration enables machines to autonomously perform experiments and optimize designs, a concept termed as self-driving laboratory (SDL) [55,[356], [357], [358], [359], [360], [361], [362]]. In UOs of 3D bioprinting, SDL methods hold promise to significantly enhance both the quantity and quality of samples, consequently reducing cost and enhancing precision, as demonstrated in previous studies [230,363].

**Fig. 10:**
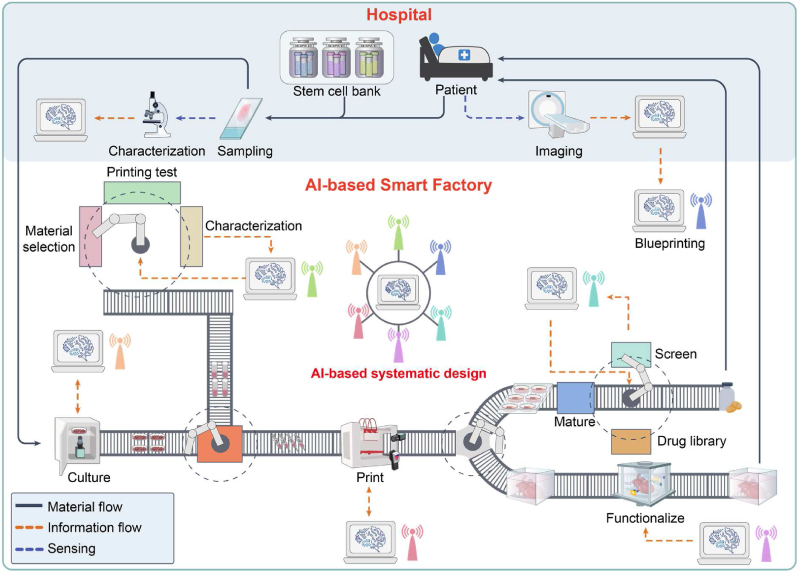
A schematic diagram of integrated automation of entire processes.

Looking ahead, the integration of AI with automation is poised to extend beyond discrete UOs to encompass entire processes (Fig. 10). The AI-based systematic design approach will unify the design of various objects, including bioinks, printed models, printing parameters, and maturation conditions. This integrated methodology will effectively consider their interdependent effects on diverse properties. For instance, through AI-driven multi-objective optimization, printed models' microstructures and bioinks' physicochemical properties can be jointly designed to meet the Pareto optimality of BPPs' mechanical properties and cell behavior. Another example can be found in 4D bioprinting, where AI can be utilized to integrate the design of printed models' microstructures, spatial distribution of bioink formulations, and external stimuli, enabling precise spatiotemporal responses of BPPs' mechanical properties [211,212,364,365].

Furthermore, the establishment of AI-based smart factories will enable efficient management of the material and information flow through advanced technologies such as industrial clouds and digital twins, facilitating full life cycle quality management, which encompasses clinical diagnosis, raw material preparation, model design, 3D printing manufacturing, and efficacy evaluation.

## 8 Conclusions

The advancement of 3D bioprinting in clinical practice confronts challenges regarding the contraction between effectiveness and economy in personalization of design, compounded by constraints in scaling-up of production due to the numerous manual operations involved. In light of these challenges, AI-driven QbD emerges as a promising solution, enhancing precision, economy, rapidity, repeatability, and scalability. This review seeks to delve into the latest advancements of AI technology applications in 3D bioprinting. Within the QbD framework, the application of AI technology in 3D bioprinting is scrutinized across three principal dimensions: multi-scale and multi-modal sensing, data-driven design, and in-line process control. Subsequently, a detailed overview of the current research status and potential applications of AI technology is provided for key elements of the 3D bioprinting process, spanning bioink formulation, model structure, printing process, and function regulation. Lastly, the development directions of AI technology in 3D bioprinting are discussed from three perspectives: construction of natural organs, active learning and hybrid learning, and integrated automation of entire processes. This comprehensive analysis aims to elucidate the potential of AI-driven approaches in catalyzing a paradigm shift in 3D bioprinting, paving the way for clinical applications.

Despite considerable progress in AI-driven 3D bioprinting, many challenges remain to be addressed. We discuss some typical challenges and their potential solutions as follows:

(ⅰ) **Scarce specificity and low quality of characterization datasets:** Regarding the characterization issues in 3D bioprinting, existing datasets are rarely tailored to the unique scenarios of this field, impeding the direct implementation of AI models trained on these datasets. For instance, in organ-scale 3D bioprinting, the precise construction of vascular networks is crucial to ensure nutrient transport and cell survival. However, in medical imaging of blood vessels, most studies focus on the retina [366], with limited datasets available for the 3D reconstruction of vascular networks in large-scale organs. Similarly, organ-scale 3D bioprinting requires a large number of cells. Yet, existing datasets for virtual staining are mostly predominantly derived from characterization results of tissue or pathological sections, with a significant lack of datasets available for non-destructive characterization of stem cells during differentiation/proliferation processes. Additionally, current datasets are typically derived from limited experimental results with narrow sources and small scales, resulting in high variability and reducing the generalization capacity of AI models. Furthermore, the performance metrics of existing AI models are often derived from independent test sets, complicating the rigorous comparison of model performance and lowering their reliability for clinical deployment.
To address this issue, ensuring the specificity and quality of datasets is imperative. Given the scale of this task, individual efforts may be insufficient, thereby encouraging collaboration among researchers in the broader 3D bioprinting community. A shared database tailored to 3D bioprinting can be established through the collection of experimental results from various research groups on cloud platforms. In this context, the specialization of the 3D bioprinting field ensures the specificity of datasets, while the diversity of research groups guarantees the multi-source and large-scale nature of datasets. Furthermore, benchmark datasets for 3D bioprinting could be established, akin to the role of ImageNet in the field of computer vision. Last but not least, during the construction of shared databases, it is crucial to address ethical, privacy, and security concerns [367,368], particularly when patients or participants are involved (such as the appropriate use of patient healthcare data). In this regard, relevant legislation should be established to regulate methods of data usage and the processes of dataset creation, accompanied by strict oversight and governance.

(ⅱ) **Insufficient universality of data-driven design for clinical deployment:** Considering the clinical translation of 3D bioprinting, patient-specific natural organs introduce a wide range of printed structures; the multi-material printing process brings numerous types of bioinks; and various bioprinter brands result in diverse 3D bioprinter configurations. This variability presents a multitude of working scenarios for data-driven design issues. While current ML models and datasets are typically built for specific scenarios, minor variations can render them unusable, requiring costly retraining.
Therefore, establishing universal ML models capable of adapting to various working scenarios will significantly reduce the need to construct datasets tailored to specific working scenarios, thereby resulting in substantial cost savings. Specifically, some design considerations are related to continuous production, such as culture conditions and printing parameters. Across different working scenarios, they follow similar process mechanisms and share relatively fixed categories of CQA, CMA, and CPP, demonstrating a certain level of universality. As an example, consider the design of printing parameters in the direct ink writing process for hydrogel-based bioinks. Across different brands of 3D bioprinters, bioink materials, and printed models, the printing process follows similar physical mechanisms, primarily the rheology of extrusion process. And the concerned CQA (such as shape fidelity of printed structure), CMA (such as rheological properties of bioinks), and CPP (such as the printing speed and extrusion speed) also remain consistent. Consequently, in theory, a universal ML model could be developed to apply across all working scenarios.
Achieving this objective necessitates the accurate identification of CQA, CMA, and CPP, accompanied by their universal and standardized descriptions, which requires a profound understanding of the process mechanism. Subsequently, the acquisition of extensive experimental data under varied working scenarios becomes imperative to construct comprehensive datasets. Ultimately, the successful development of universal ML models hinges upon meticulous architectural design and training methods predicated on the data structure of the inputs/outputs. To summarize, expertise in 3D bioprinting and AI is required to ensure accurate predictions across diverse working scenarios.

(ⅲ) **Limited consideration of spatiotemporal dynamics in ML models:** With the development of 3D bioprinting technology, the four key elements in this field have increasingly exhibited spatiotemporal dynamics. Firstly, with the advent of dynamic hydrogels, the implanted BPPs undergo multi-scale dynamic interactions with the in vivo environment. At the macro scale, the degradation dynamics of BPPs should align with the host remodeling of the construct; while at the micro scale, the microenvironment provided by hydrogels regulates cell behaviors over time. Similarly, the emerging 4D bioprinting focuses on the stimuli-responsive shape morphing of printed structures over the additional temporal dimension. Additionally, in-situ bioprinting is expected to accommodate the spatiotemporal changes of printing surfaces. In summary, these spatiotemporal dynamics present significant challenges to the design and manufacturing of 3D bioprinting, yet most existing AI models are still confined to static scenarios.
In this context, ML models excelling at handling sequential data, such as RNN, LSTM, GRU, and Transformer, demonstrate strong temporal modeling capabilities, offering substantial potential for effectively managing these spatiotemporal dynamics.

Addressing the above-mentioned challenges will significantly advance the authentic development of human organ substitutes, facilitating the translation of 3D bioprinting from bench to bedside.

## CRediT authorship contribution statement

**Zhenrui Zhang:** Writing – review & editing, Writing – original draft, Visualization, Validation, Software, Methodology, Investigation, Formal analysis, Data curation, Conceptualization. **Xianhao Zhou:** Writing – review & editing, Writing – original draft, Visualization, Validation, Software, Methodology, Investigation, Formal analysis, Data curation, Conceptualization. **Yongcong Fang:** Validation, Supervision, Resources, Project administration, Funding acquisition, Formal analysis, Conceptualization, Writing – review & editing. **Zhuo Xiong:** Validation, Supervision, Resources, Project administration, Funding acquisition, Formal analysis, Conceptualization. **Ting Zhang:** Validation, Supervision, Resources, Project administration, Funding acquisition, Formal analysis, Conceptualization.

## Ethics approval and consent to participate

The current review does not involve any experimental work on human subjects or animals, and thus does not require approval from an ethics committee. As this work solely involves the synthesis and analysis of pre-existing literature, it doesn't necessitate any form of direct involvement or consent from patients or healthy volunteers.

## Declaration of competing interest

We declare that we have no financial and personal relationships with other people or organizations that can inappropriately influence our work, there is no professional or other personal interest of any nature or kind in any product, service, and/or company that could be construed as influencing the position presented in, or the review of, the manuscript entitled, “Artificial intelligence-driven 3D bioprinting for regenerative medicine: From Bench to Bedside".
